# Inversion in the permeability evolution of deforming Westerly granite near the brittle–ductile transition

**DOI:** 10.1038/s41598-021-03435-0

**Published:** 2021-12-15

**Authors:** Claudio Petrini, Claudio Madonna, Taras Gerya

**Affiliations:** 1 Department of Earth Sciences, Institute of Geophysics, ETH Zurich, Zürich, Switzerland; 2 Department of Earth Sciences, Geological Institute, ETH Zurich, Zürich, Switzerland

**Keywords:** Geophysics, Tectonics, Hydrogeology, Structural geology

## Abstract

Fluid flow through crustal rocks is controlled by permeability. Underground fluid flow is crucial in many geotechnical endeavors, such as CO_2_ sequestration, geothermal energy, and oil and gas recovery. Pervasive fluid flow and pore fluid pressure control the strength of a rock and affect seismicity in tectonic and geotechnical settings. Despite its relevance, the evolution of permeability with changing temperature and during deformation remains elusive. In this study, the permeability of Westerly granite at an effective pressure of 100 MPa was measured under conditions near its brittle–ductile transition, between 650 °C and 850 °C, with a strain rate on the order of 2·10^–6^ s^−1^. To capture the evolution of permeability with increasing axial strain, the samples were continuously deformed in a Paterson gas-medium triaxial apparatus. The microstructures of the rock were studied after testing. The experiments reveal an inversion in the permeability evolution: an initial decrease in permeability due to compaction and then an increase in permeability shortly before and immediately after failure. The increase in permeability after failure, also present at high temperatures, is attributed to the creation of interconnected fluid pathways along the induced fractures. This systematic increase demonstrates the subordinate role that temperature dilatancy plays in permeability control compared to stress and its related deformation. These new experimental results thus demonstrate that permeability enhancement under brittle–ductile conditions unveils the potential for EGS exploitation in high-temperature rocks.

## Introduction

The global demand for cleaner energy, such as geothermal energy, is booming, but the high investment costs associated with subsurface operation still inhibit the realization of projects at the industrial scale (e.g., Finger and Blankenship^1^ and Rossi et al. ^2^) To date, only shallow high-temperature regions located in the vicinity of a magma body (< 2 km) (e.g., Elders et al.^3^, Scott et al.^4^, and Watanabe et al.^5^) have been exploited to generate electricity, while deeper regions remain a target. Most tectonic settings require drilling deeper than 5 km to achieve the conditions needed to operate a power plant. At these depths, the mechanical and transport properties of geothermal rocks are difficult to measure or predict, introducing large uncertainties in the design of a geothermal field. One such property is permeability.

Permeability is a second-order tensor that describes the ability of porous media to transmit fluids^6^. Several studies have focused on measuring the permeability of rocks (e.g., Bernabe et al. ^7^, Brace et al.^8^, Selvadurai and Głowacki^9^, Wenning et al.^10^, Bernabe^11^, and Bakker et al.^12^). However, only a few laboratory studies have described how this parameter evolves under high temperatures, > 350 °C, and continuous deformation^5,13,14^. Knowledge of the permeability evolution under such transient conditions is an important prerequisite for predicting the behavior of a geological reservoir, particularly when production and/or injection takes place. Percolation of pressurized fluids along critically stressed faults can trigger seismicity (e.g., Miller et al.^15^), in not only enhanced geothermal systems (EGSs)^16^ but also many natural tectonic settings. In this context, a better understanding of the stress and temperature dependency of permeability is needed.

The temperature dependency of permeability has been investigated on only a limited scale, and contrasting trends in the evolution of permeability were obtained depending on the investigated rock type (e.g., igneous, metamorphic, and sedimentary)^17^. A positive correlation between temperature and permeability was attributed to the formation of microcracks between different minerals due to the different thermal expansion coefficients in several rock types (igneous and metamorphic)^18^. On the other hand, Shmonov et al.^17^ observed an initial drop in permeability with increasing temperature, with a later increase above 300 °C. In follow-up studies, they observed a consistent decrease in permeability with increasing temperature^17^; similarly, Bakker et al.^12^ showed a permanent reduction in the permeability of limestone with increasing temperature. The observed decrease was attributed to the ductile closure of the initial pore space by dislocation creep of the minerals due to viscous relaxation induced by thermoelastic stresses, pressure solubilization, or an “excess” of thermal expansion of the rock^18^.

Deformation plays a leading-order contribution to the evolution of permeability and the fluid’s percolation behavior. Mitchell and Faulkner^19^ studied the permeability of Cerro Cristales granodiorite and Westerly granite at room temperature, between 10 and 50 MPa effective pressure, and under an increase in differential stress, recording increases in permeability up to two orders of magnitude. This increase was higher before macroscopic failure of the specimen, mainly due to the formation of microfractures. In contrast to materials that exhibit higher initial porosity, Suri et al.^20^ investigated permeability changes in Indiana limestone under increasing differential stress and recorded a decrease in permeability due to pore collapse with increasing deformation. Investigations on the effect of brittle and ductile deformation on anhydrite at room temperature revealed an increase of up to two orders of magnitude for both brittle and ductile deformation^21^. In this study, pore volume changes were monitored during the experiments to infer an increase in permeability with decreasing effective pressure.

Several other studies have investigated the influence of high-temperature conditions on permeability evolution. Fischer and Paterson^22^ measured the permeability of three different rock types (limestone, marble, and sandstone) in a Paterson gas-medium triaxial apparatus at confining pressures up to 300 MPa, pore fluid pressures up to 250 MPa and temperatures exceeding 600 °C. The oscillation method was employed to capture changes in permeability due to increasing differential stresses at different stress intervals. Zhu and Wong^23^ carried out investigations on sedimentary rocks at room temperature, measuring their permeability during axial deformation, and stated that independent of brittle faulting or cataclastic flow, permeability decreases with an increase in the effective mean stress, which contradicts previously reported increases in permeability, especially before brittle failure and in low-porosity rocks (e.g., Mitchell and Faulkner^19^ and De Paola et al.^21^). The decrease in permeability described by Zhu and Wong^23^ was concluded to be related to a variable behavior between low- and high-porosity rocks when subjected to a stress increase. Low-porosity rocks tend to exhibit an increase in permeability with failure; conversely, the pore space of high-porosity rocks becomes more tortuous due to microcracking^23^. On the other hand, studies carried out on crystalline rocks clearly show an increase in permeability with increasing deformation^19,24,25^. For example, Coelho et al.^25^ carried out permeability measurements of altered basalts in a Paterson apparatus at pressures and temperatures of 100 MPa and 400 °C, respectively, recording the permeability at different steps before and after the peak differential stress point of the samples, highlighting an increase in permeability after sample failure. Zoback and Byerlee^24^, on the other hand, showed a positive correlation with increasing differential stress by investigating the effect of porosity variation on the permeability evolution of Westerly granite under differential stress.

Permeability measurements at high pressures and temperatures are undoubtedly still difficult to perform^26^. Therefore, for measurements under continuous deformation, permeability is mostly inferred from continuous porosity measurements (e.g., Violay et al.^13^). However, the conversion between porosity and permeability can be ambiguous. While the former is a scalar describing the pore volume, the latter can be independent of pore volume or only partially associated with it, and this makes a direct translation between the two unreliable—particularly when more deformation modes coexist, such as brittle and ductile deformation^27^. Notably, regions characterized by brittle–ductile deformation can exist near the Earth’s surface and may be of potential interest for EGS exploitation (e.g., Watanabe et al.^5^, Violay et al.^13^, and Noël et al.^27^). To obtain a more comprehensive understanding of how permeability evolves throughout deformation and under conditions that feasibly represent shallow, high-temperature rocks with a potential for geothermal exploitation, additional experiments are needed to evaluate the important relationship between high-temperature-induced deformation and the continuously evolving permeability to a higher degree of certainty.

In this study, we present effective permeability measurements of fine-grained granite at an effective pressure of 100 MPa, under the assumption of a simple effective pressure law^28,29^, and a temperature up to 850 °C under continuous axial deformation in a Paterson gas-medium triaxial apparatus^30^. The carefully conducted experiments show that the permeability enhancement of granite is possible during deformation at strain rates on the order of 2·10^−6^ s^−1^. Our results show that at high temperatures, the main mechanism controlling permeability enhancement is the creation of new fluid pathways during deformation, while temperature dilatancy is subordinate. This also confirms that a change in permeability is not necessarily related to a change in porosity.

The “Results” section describes the experimental data, the “Discussion” section analyzes the findings and the main implications of this study and outlines future research directions, and the “Methods” section at the end of the manuscript describes the sample preparation and properties and the experimental procedure.

## Results

**Figure 1:**
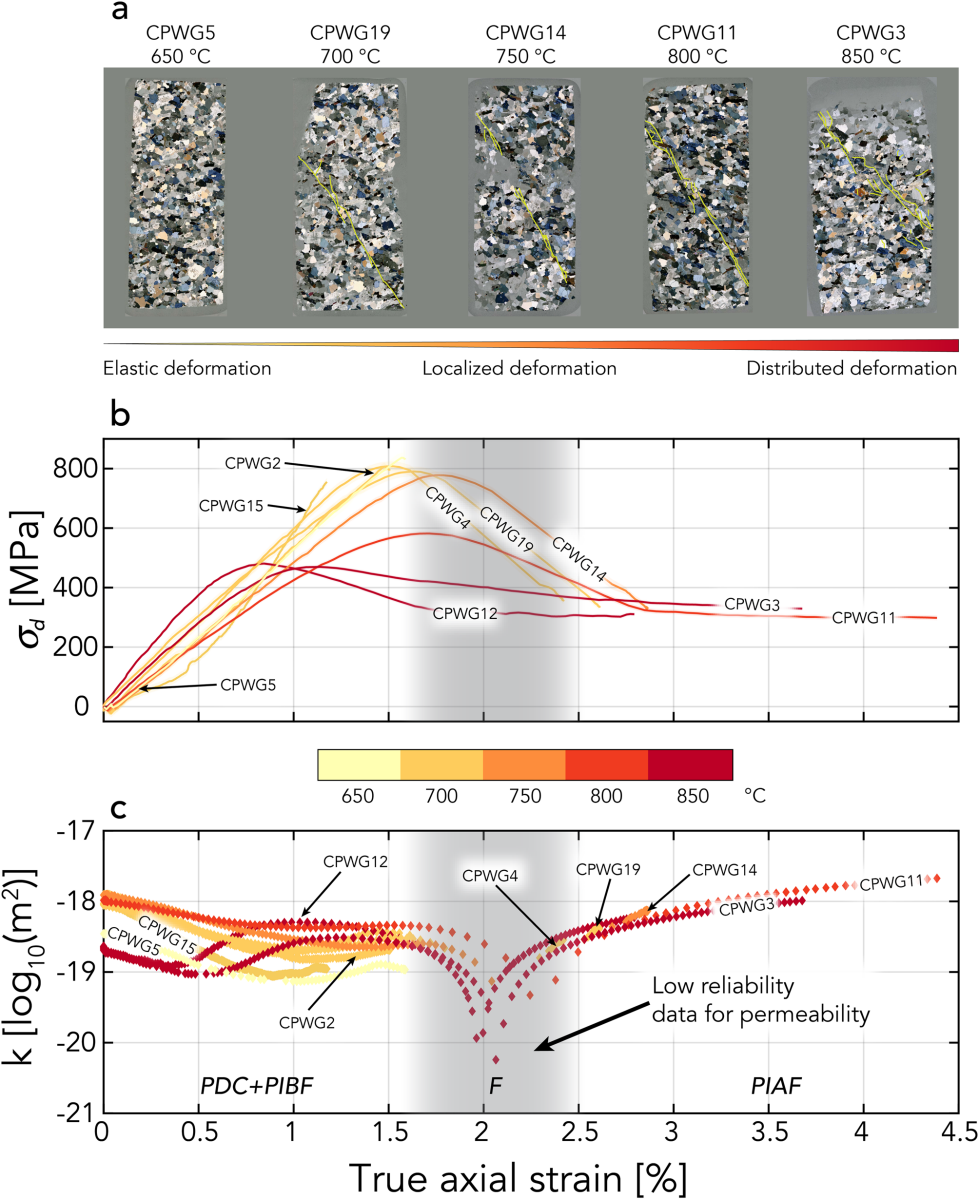
(**a**) Optical microscopy images of thin sections under cross polarized light of the deformed rock, with the major fault plane marked in green. (**b**) Stress–strain curves for the different samples at different temperatures show an increase in ductile behavior with increasing temperature. (**c**) Permeability evolution of each tested specimen from 650 °C to 850 °C. The permeability and differential stress errors are given in Table 1. *PDC*: permeability decrease by compaction; *PIBF*: permeability increase before failure; *F*: Failure; *PIAF*: permeability increase after failure.

For simplicity and clarity, only data for each experimental condition (CPWG5, CPWG19, CPWG14, CPWG11, and CPWG3) are analyzed and discussed in the following sections. Nevertheless, the permeability measurements and mechanical data for all the tested samples are represented in Fig. 1 and Table 1.

**Table 1:** Experimental results for all the tested samples.

Sample	Initial permeability [m^2^]	Mean permeability error [%]	Peak differential stress [MPa]	Mean stress error [%]	Strain at peak differential stress [%]	Failure mode
ETHZ_2016_CPWG5	3.48·10^–19^	4.07	843	3.53	–	–
ETHZ_2016_CPWG2	1.24·10^–18^	4.07	807	3.80	1.48	Localized shear failure
ETHZ_2016_CPWG4	1.06·10^–18^	4.20	808	3.40	1.48	Localized shear failure
ETHZ_2016_CPWG15	9.05·10^–19^	4.05	756	5.77	1.18	Localized shear failure
ETHZ_2016_CPWG19	9.21·10^–19^	4.12	791	3.53	1.64	Localized shear failure
ETHZ_2016_CPWG14	1.22·10^–18^	4.15	778	2.84	1.74	Localized shear failure
ETHZ_2016_CPWG11	1.03·10^–18^	4.19	583	2.23	1.72	Localized shear failure
ETHZ_2016_CPWG3	2.18·10^–19^	4.95	470	1.73	1.11	Distributed shear failure
ETHZ_2016_CPWG12	2.29·10^–19^	5.16	481	1.85	0.84	Distributed shear failure

Figure 1 shows the thin sections and the permeability and stress evolution as a function of the true axial strain of the tested specimens. The shaded boxes in Fig. 1b,c represent the fast rupture stage of the sample, where the reliability of permeability values is low because of method limitations in measuring permeability at high strain rates. The range of low data reliability is slightly different for each sample due to its strain rate dependency. To account for this variability and for display purposes, a shaded boundary is shown. For consistency, these data are plotted but are not considered in the interpretation of the experimental results. The initial permeability for samples deformed between 700 °C and 800 °C lies between 9·10^–19^ m^2^ and 1.2·10^–18^ m^2^; for samples measured at 650 °C and 850 °C, the initial permeability is lower, 3.48·10^–19^ m^2^ for sample CPWG5 at 650 °C, and approximately 2.2·10^–19^ m^2^ for the samples measured at 850 °C, CPWG3 and CPWG12 (Table 1). The overall change in permeability as a function of the true axial strain is represented by an increase of up to half an order of magnitude at both high pressure and temperature (Fig. 1). The change in permeability is, however, not uniform with axial deformation, and its increase is most significant after specimen failure. Furthermore, a general decrease in the maximal differential stress correlates with an increase in the temperature.

### Postexperiment thin section analysis

Figure 1a shows thin sections retrieved after the experiments. The petrographic analyses and the stress–strain curves of the specimens from experiments under temperatures from 700 °C to 850 °C show a shear failure dominated deformation, with a prevalent brittle deformation mode (Fig. 1a,b). With increasing temperature, the single well-localized fracture plane that developed in experiments up to 750 °C transitions to a conjugated system of fractures. With increasing temperature, the conjugated system of fractures widens and becomes broader, indicating that a ductile component in the deformation was present and increasingly important. From 750 °C to 850 °C, the system of fractures gradually became a distributed cluster of cracks instead of a single rupture. In addition to a broad fracture cluster, sample CPWG3, deformed at 850 °C, undoubtedly shows at macroscopic scale a barreling effect, which is typically found in experimentally ductile-deformed rocks (Fig. 1a). Moreover, the peak differential stress decreases with temperature, which is another indicator of ductile deformation.

### Stress–strain and permeability analysis

At 650 °C, the peak differential stress, at which failure occurs, was not reached because of the technical limitations of the load cell (Fig. 1b). Up to 1% true axial strain, the samples underwent a permeability reduction of almost an order of magnitude (Fig. 1c). With a further increase in true axial strain, permeability increased up to slightly less than half an order of magnitude from its lowest value until the maximal experimental stress was reached.

The 700 °C experiment corresponds to a peak differential stress of approximately 800 MPa. The permeability decreased up to an approximately 1% true axial strain and subsequently increased immediately before the peak differential stress, similar to the 650 °C experiment (Fig. 1c). However, this behavior is less pronounced than in the specimen measured at 650 °C. After rupture, a permeability almost half an order of magnitude higher than the lower permeability reached during compaction was measured.

The peak differential stress at 750 °C was slightly lower but similar in magnitude to that at 700 °C. The permeability evolution displayed a decrease, as for previous specimens; however, the increase before failure was significantly lower in magnitude. After rupture, an increase in permeability of half an order of magnitude was measured, similar to the 700 °C experiment.

At 800 °C, the peak differential stress was decreased by approximately 200 MPa compared to its value under the lower temperatures. The change in permeability was similar to that exhibited in the 750 °C experiment. After initial compaction, the permeability remained nearly constant. The 800 °C experiment exhibited an increase in permeability only after the sample reached its peak differential stress, i.e., after approximately 2.5% true axial strain. With an increase in the true axial strain, the permeability increased significantly, by more than half an order of magnitude (Fig. 1c).

The experiment carried out at 850 °C shows the lowest recorded maximal differential stress of all our experiments (~ 450 MPa) (Fig. 1a,b). The permeability evolution with respect to the true axial strain recorded at 850 °C exhibited a more complex behavior than the lower temperature measurements (Fig. 1c). The 850 °C experiment exhibited a marked decrease in permeability during the first 0.5% of true axial strain and immediately afterward exhibited an increase in permeability of an equivalent magnitude. After reaching the peak differential stress point, the permeability increased by more than half an order of magnitude with an increase in the true axial strain, similar to that in the 800 °C experiment.

### Scanning electron microscopy (SEM) thin section analysis

**Figure 2:**
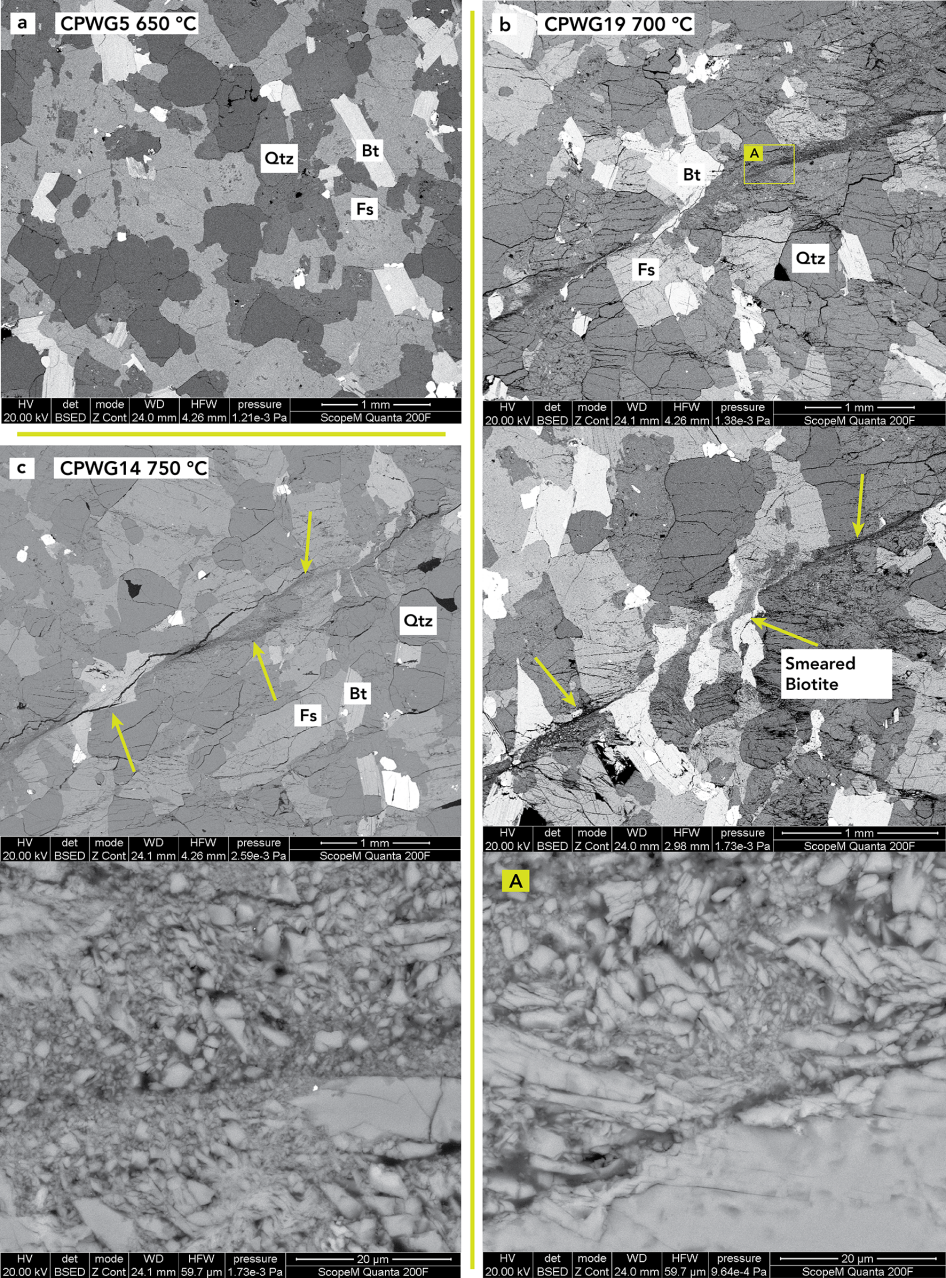
SEM images of a thin section after deformation at 650 °C, 700 °C, and 750 °C. (**a**) CPWG5 at 650 °C, (**b**) CPWG19 at 700 °C, and (**c**) CPWG14 at 750 °C. The CPWG5 specimen shows no brittle failure or undeformed grains, because the sample did not reached failure and experienced a much smaller strain compared to the other tested specimens. The 700 °C image shows a major shear zone with a damaged zone around it. The strain is mainly accommodated by brittle deformation and partly by smeared out Biotite. The sample that deformed at 750 °C also shows a major shear zone with some deformation around it and an important grain size reduction in the fault gouge, similar to the deformation at 700 °C (magnified green box A). The green arrows indicate the shear zones and cracks.

**Figure 3:**
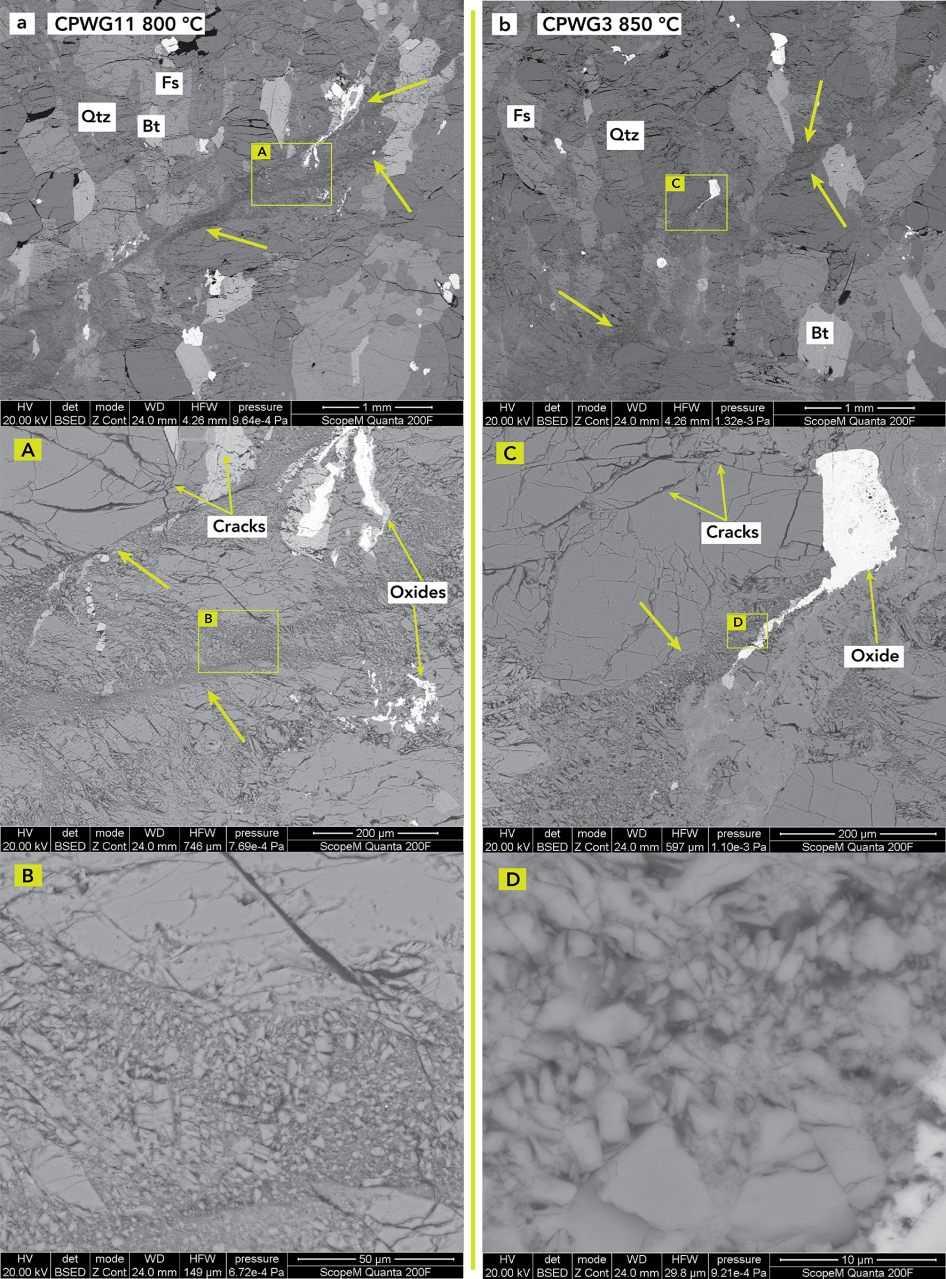
SEM images of thin sections after deformation at 800 °C and 850 °C. (**a**) CPWG11 at 800 °C and (**b**) CPWG3 at 850 °C. Deformation at 800 °C presents different shear planes, with highly damaged regions, seen in the magnified green boxes A and B. At 850 °C, the shear planes are less evident, and the deformation is less localized, with a highly damaged central zone that has a region with small grain sizes (magnified green boxes C and D). The fault gouge is evidence of cataclastic flow and oxides deforming in a ductile way in both specimens. The green arrows indicate the different shear zones and cracks that characterize the deformation as more ductile.

Analysis of the resulting deformation structures can further help understand and confirm the mechanisms inferred from the permeability curves. All the samples deformed above 650 °C ruptured and showed fracturing in the postexperiment analysis (Figs. 2 and 3). SEM images show, in detail, the variations in the fracture patterns formed in the thin sections at the different experimental temperatures (Figs. 2 and 3).

At 650 °C, the sample did not fail under the axial load; the decrease in permeability and its subsequent increase before failure (Fig. 1) can be related to only matrix deformation (Fig. 2). Thin section SEM imaging of the specimen after deformation (Fig. 2) displays no visible fracture or evident grain deformation indicating any specific deformation mechanism, which suggests that the permeability evolution was mainly related to the macroscopic elastic and subgrain size matrix deformation.

Specimens that underwent rupture at 700 °C and 750 °C (Fig. 2) behaved similarly and showed a single main fracture plane with no significant damage zone around the fault core. Inside the main fault zone, a narrow region with grain size reduction, where most of the deformation is accommodated, can be seen (Fig. 2). The main deformation mechanism is cataclastic flow and brecciation of the initial grains (Fig. 2, magnified green boxes). Within the same fault zone, the ductile deformation of biotite accommodated some strain as well (Fig. 2). The fracture created a well-defined pathway for the fluid, which enhanced the permeability after failure.

At 800 °C, the main deformation zone was less sharp than those that formed at lower temperatures, and multiple secondary ruptures formed a cluster of fractures (Fig. 3). Furthermore, around the main fracture, a wider damaged zone formed a region of smaller grains, defining the deformation zone at 800 °C. The main deformation mechanism of the fracture was cataclastic flow, resulting in a very small grain size in the fault gouge (Fig. 3; magnified green boxes A and B). Furthermore, Fig. 1a,b shows that the shear failure of the specimen is accompanied by a ductile deformation component. Additionally, Fig. 3 shows that in addition to the main brittle deformation mechanism along the fault, the ductile deformation of biotite and oxides accommodates some deformation.

At 850 °C, the deformation was more distributed, with many secondary fractures and a broad region of highly damaged and crushed grains (Fig. 3). When compared to the rupture patterns at low temperature, the fault gouge was thicker and more pronounced than at 800 °C (Figs. 1a and 3), which indicates that the deformation had a ductile component (Fig. 1a,b) and that the tortuosity of the fluid paths was possibly greater. The fracture gouge shows a significant grain size reduction with crushed grains, again indicating cataclastic flow as the main deformation mechanism (Fig. 3 magnified green boxes C and D). The cataclastic flow in the main fracture zone was accompanied by the plastic deformation of oxides and minerals (Fig. 3; magnified green boxes A and C).

## Discussion

Permeability can be influenced by many factors, such as the stress distribution, deformation rate, fracture orientation, geometry, connectivity, tortuosity, and aperture, or rock mineral anisotropy. Due to the multifaceted nature of permeability, the location, duration, and conditions of experiments may have both strong and variable implications. In this study, the permeability measured represents a bulk measurement, which describes the permeability of a sample parallel to the maximal principal stress axis. The variability in initial permeability and the subsequent evolution, as well as the small differences among the mechanical results of our tests, can be related to the spatial variability of the microstructures (porosity, microcrack distribution, microcrack connectivity, etc.) present in the rock prior to deformation. Additionally, the different experimental target temperatures could influence the starting microcrack distribution, leading to different initial values^13^ and consequently small differences in the observed permeability evolution and sample deformation. Nevertheless, the initial permeability values recorded in our study are in good agreement with the permeabilities proposed for Westerly granite in Violay et al.^13^ and Mitchell and Faulkner^19^. The elastic deformation is well in the range of the previous observations at all the tested temperatures and differs only slightly. The variations recorded among the elastic deformations can also be attributed to a different initial population of microcracks, due to different experimental temperatures, and to initial sample heterogeneities^31,32^.

**Figure 4:**
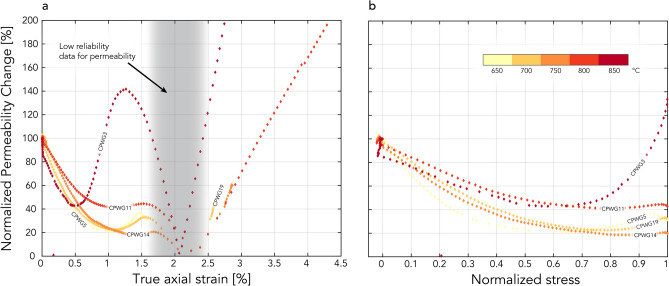
Normalized permeability evolution. (**a**) Normalized permeability change compared to true axial strain, (**b**) normalized permeability compared to normalized differential stress. For display purposes, the y-axes were limited up to a permeability increase of 200%, and in (**b**), only the data recorded before the peak differential stress are shown. The permeability is normalized by the initial permeability measured prior to deformation, whereas the differential stress is normalized by the peak differential stress. The region of data with low reliability is represented as in Fig. 1.

Permeability values during the fast failure stage of the experiments (shaded boxes in Figs. 1b,c, and 4a) cannot be determined with accuracy because of the high strain rates acting during this rapid deformation phase. The high strain rates during the fast failure stage of a sample imply that the frequency and amplitude used for our permeability measurements are unable to capture the fast permeability changes with a reliable number of oscillations, precluding accurate permeability measurements. Furthermore, due to the high strain rates during failure, drained conditions are not ensured during this phase of deformation, preventing any reliable permeability values from being measured throughout this stage.

The permeability evolutions recorded in our experiments show similar behavior before and after failure (Fig. 4) and are analogous to those of the experiments of Mitchell and Faulkner^19^ carried out at room temperature. Nevertheless, in our experiments, a decrease and then increase in permeability was observed before the peak differential stress (Fig. 4a,b). The increase was lower in magnitude than that presented in Mitchell and Faulkner^19^ at room temperature, and the magnitude of increase decreased with increasing temperature up to 800 °C. The permeability changes up to 750 °C follow a similar trend to the porosity evolution recorded at the same effective pressure and similar temperatures by Violay et al.^13^ on the same rock type. Likewise, the initial reduction in permeability is attributable to compaction, i.e., the pore and microcrack closure correlates well with an increase in differential stress (Fig. 4b). The permeability increase reached before the peak differential stress can be attributed to microfracturing induced by axial deformation and their interconnection, which eases fluid flow (Figs. 2 and 3). At higher temperatures, microfracturing decreases^33^, and the increase in permeability before the peak differential stress decreases (Fig. 4a,b). This is possibly attributed to dislocation motion of the grains themselves^33^, as well as a broader distribution of the unlinked microcracks (Fig. 3), which potentially inhibit fluid flow more in the high-temperature cases than in the low-temperature cases, where the microcracks are more concentrated and interconnected close to the main fault plane (Figs. 2 and 3). For the experiments up to 750 °C, the postfailure permeability enhancement is attributed to shear deformation along the fault, where shearing of the fault may produce an increase in the fracture aperture and thus in permeability (e.g., Crawford et al.^34^). This creates fluid paths that facilitate flow (Fig. 2).

The permeability evolution with increasing axial strain at 800 °C follows the same trend as in that observed in the lower temperature experiments. A reduction in porosity does not necessarily correlate with a decrease in permeability since permeability can also be enhanced by the creation of new fluid paths induced by fracture creation. This mechanism is, to a certain degree, independent of pore volume changes and allows permeability enhancement under compaction conditions, where a decrease in porosity is observed^13^. Similar behavior can be seen in the experiment performed at 850 °C, where permeability also increases after failure. Compared to the other experiments, the permeability behavior at 850 °C is more complex. The marked increase in permeability at approximately 0.5% axial strain, before the peak differential stress (Fig. 4b), is attributed to the beginning of the sample failure rather than to dilatancy. Dilatancy effects are probably not present or are small in magnitude at such high temperatures, as suggested by Violay et al.^13^, where above 800 °C, no increase in pore volume is observed. The prominent increase in permeability before the peak differential stress is associated with the macroscopic shear failure process of the sample and is directly related to an earlier transient phase characterized by microcracking, typical for brittle–ductile deformation, necessary to reach the macroscopic shear failure (Figs. 1 and 3)^27^. The increase in permeability before and after macroscopic failure shows the same slope (Fig. 4a), thus suggesting that the increase before and after macroscopic failure can be related to the same brittle–ductile deformation process.

The data presented in Figs. 1b,c, and 4 show that the strain has a greater influence on the evolution of permeability than the increasing experimental temperature. The influence of temperature is primarily observed in the change in deformation mode and mechanisms, which in turn then affect the permeability evolution. An additional aspect that can influence the microstructures and permeability is the extent of prolonged shearing on the failure plane that the samples undergo after shear failure. Samples CPWG11 (800 °C) and CPWG3 (850 °C) were subjected to substantially different true axial strains, which could eventually lead to differences in microstructures compared to the other samples. However, by comparing the data of CPWG3 and CPWG12 (both run at 850 °C) presented in Fig. 1, it is evident that the main permeability changes occur before a true axial strain of approximately 2.5%, i.e., before the main macroscopic failure ends in the sample. The prolonged shearing of CPWG3 did not produce an important increase in permeability compared to the less sheared CPWG12. From such data, we deduced that the main microstructures influencing permeability are created before a true axial strain of approximately 2.5% and that further shearing will not significantly affect these microstructures and the related permeability.

Although all our experiments were run under fully drained conditions, it cannot be excluded that during deformation, isolated zones of high fluid pressure form locally in the samples, thus decreasing their strength to some degree (cf., e.g., Petrini et al.^35^, Faulkner and Rutter^36^, Farquharson et al.^37^, and Fig. 1b). Based on the model presented in Petrini et al.^35^, this high fluid pressure inside the sample is caused by locally more efficient viscoplastic compaction during shear localization.

While we cannot directly relate permeability to porosity, we can estimate that the porosity changes did not differ significantly from those displayed in Violay et al.^13^, where Westerly granite was also investigated. By comparison, permeability is only partially dependent on porosity evolution, particularly at high temperatures close to the ductile regime. An increase in permeability still arises at high temperatures where pore closure is recorded^13^, indicating that the mechanisms governing permeability evolution are related to not only pore space but also other mechanisms. For example, grain crushing and the consequent interconnected fluid pathway formation need to be investigated more carefully.

The limited independence of permeability from pore volume evolution indicates that fluid pathways can be created and sustained in partially compacting rocks characterized by a ductile deformation component. Although the values measured in our experiments are slightly below the critical value necessary to potentially exploit geothermal energy (i.e., approximately 1·10^–17^–1·10^–15^ m^2 ^^5,6^), we can infer that in reservoir rock with a fairly low transition temperature, such as Westerly granite^5^, permeability enhancement can be achieved and sustained. This may justify the exploitation of geothermal resources in high-temperature reservoir rocks. Despite prevalent compacting conditions in such settings, fluid pathways are potentially sufficient to create fluid circulation under conditions where rocks behave in a brittle–ductile manner. An increase in permeability was also seen at high temperatures in hydraulic fracturing experiments carried out on granite^38^, which supports our interpretation. Under more ductile conditions, fractures created by injection are smaller than those created under brittle conditions, but their number is higher and their distribution around the injection hole is much broader, creating a fracture network able to significantly enhance permeability^38^. A fairly similar fracture pattern behavior is also observed in our experiments, where experiments on samples at high temperature with an increasing distributed shear failure exhibit a higher fracture density and distribution around the main fault plane (Figs. 1a and 3). This confirms the increase in permeability at high temperatures, where pore volume increases are usually not observed^13^.

A more extensive understanding of the permeability behavior of rocks under dynamic conditions is crucial for not only performing reservoir-scale environments but also elucidating pervasive fluid flow in large-scale tectonic environments, such as subduction zones (e.g., Petrini et al.^35^) or seismically active faults (e.g., Miller et al.^15^). Permeability, and thus fluid flow, is known to have an important impact on the mechanics of subduction thrust seismicity (as well as induced seismicity, e.g., Keranen and Weingarten^39^). In geodynamic investigations, only reasonably simplistic permeability evolution laws are used. Such laws usually include a direct permeability dependence on porosity but no other mechanism (e.g., Carman^40^, Connolly and Podladchikov^41^, and Morency et al.^42^). Including additional information and mechanisms on how permeability evolves under dynamic conditions from the results of laboratory experiments would significantly contribute to further fluid transport investigations.

All the Westerly granite test specimens that were subjected to deformation at high pressures and temperatures exhibited a decrease and then increase in permeability before ultimate failure. The nature of such permeability changes can be related to (i) the closure of pores and microcracks during the early stages of deformation and (ii) pore volume increases due to associated microfracturing and dilatancy as the samples approach failure at temperatures below 700 °C. The magnitude of the permeability change with increasing temperature decreases at temperatures above 700 °C due to an increase in the ductility of the deforming rock (Fig. 1). A permeability enhancement was observed in all specimens after macroscopic failure and was caused by the creation of interconnected fluid pathways along fractures during the failure process. Such pathways resulted in the channeling of fluid and facilitated its pervasive flow through the sample. Furthermore, we showed that permeability enhancement can be partially independent of pore volume changes because newly created fluid pathways can overcome the compacting processes that are typically observed in rocks at high temperatures.

The results of this study corroborate previous efforts studying the relationship between pore volume changes and permeability (e.g., Violay et al.^13^ and Mitchell and Faulkner^19^) and provide new measurements of permeability as rocks deform at the brittle–ductile transition. The possibility of enhancing permeability under brittle–ductile conditions could significantly reduce seismicity (e.g., Asanuma et al.^43^, Tsuchiya et al.^44^, and Sibson^45,46^) while ensuring a permeability high enough to create a geothermal reservoir. This study suggests that at high temperatures, permeability enhancement in a brittle–ductile deformation regime (Fig. 1) is feasible, offering new insights into rock-fluid mechanics, with important implications for the realization of future EGSs.

## Methods

### Experiment preparation

Westerly granite was used in this work due to its fine-grained (0.05–2.2 mm), isotropic, and homogenous texture^47–49^, which is well suited for experiments where sample dimensions are relatively small. Westerly granite is a low-porosity rock (~ 0.8%) and has a low permeability (approximately 1·10^–18^–1·10^–19^ m^2^)^13,19^. Since Westerly granite is considered to be isotropic in terms of its permeability and mechanical properties, no special care had to be taken for representative sample selection during coring. Several specimens of approximately 10 mm diameter, with a length of approximately 20 mm, were cored from a larger specimen (Table 2). The sample length-to-diameter ratio of 2:1 was used to avoid boundary effects on the stress distribution and strain partitioning^50^. Each core was then cut with a precision saw to the exact length, and the end planes were ground using a lathe to ensure that they were smooth and parallel. The samples were stored in an oven without vacuum at 60 °C for at least 1 week before the experiment to reduce the initial moisture content.

**Table 2:** Summary of the experimental conditions used for the permeability experiments for each tested rock specimen.

Sample	Porosity [%]	P_confining_ [MPa]	P_fluid_ [MPa]	P_effective_ [MPa]	Temperature [°C]	Mean strain rate [s^−1^]
ETHZ_2016_CPWG5	0.770	150	50	100	650	1.7·10^–6^
ETHZ_2016_CPWG2	0.906	150	50	100	700	9.7·10^–7^
ETHZ_2016_CPWG4	1.114	150	50	100	700	1.6·10^–6^
ETHZ_2016_CPWG15	0.909	150	50	100	700	4.9·10^–7^
ETHZ_2016_CPWG19	0.827	150	50	100	700	1.7·10^–6^
ETHZ_2016_CPWG14	0.789	150	50	100	750	1.9·10^–6^
ETHZ_2016_CPWG11	0.762	150	50	100	800	3.4·10^–6^
ETHZ_2016_CPWG3	0.640	150	50	100	850	2.4·10^–6^
ETHZ_2016_CPWG12	0.612	150	50	100	850	1.9·10^–6^

### Sample assembly

**Figure 5:**
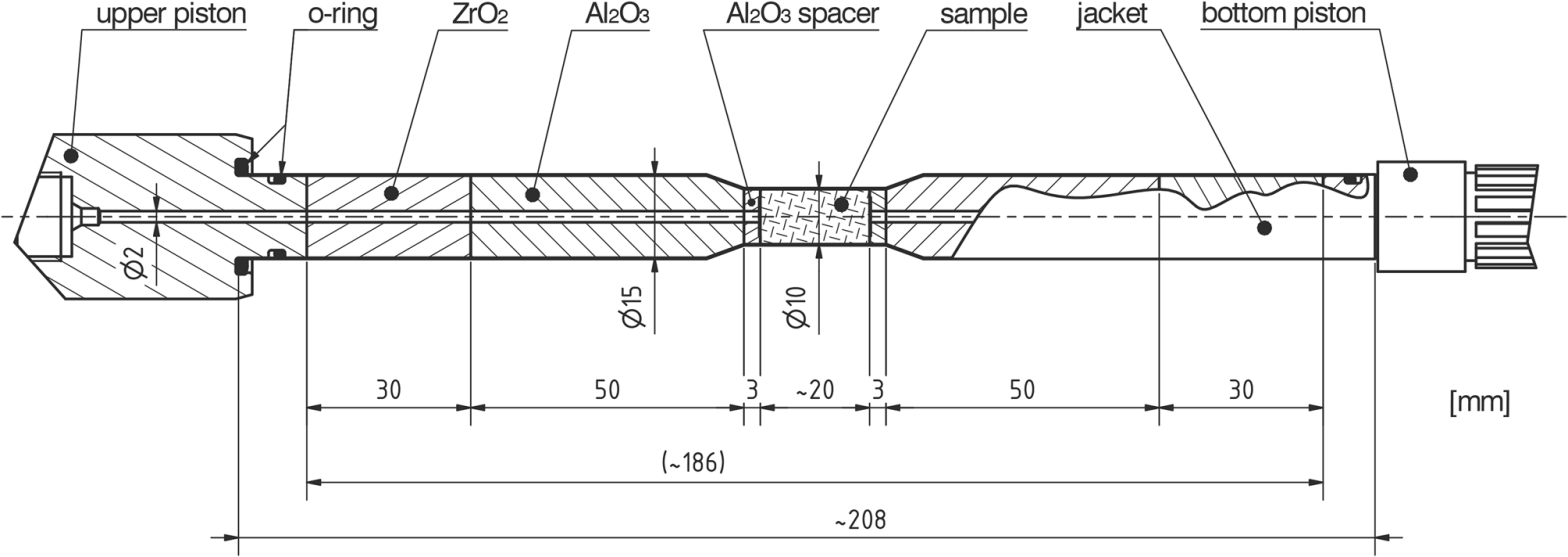
Schematic sample assembly drawn after Fischer and Paterson^22^ (image courtesy of Thomas Mörgeli).

An alumina (Al_2_O_3_) spacer, alumina piston, and zirconia (ZrO_2_) piston were set in sequence from the sample to the upper and lower steel pistons (Fig. 5). All pistons and spacers had a 2 mm hole in the center that allowed the fluid, argon, to reach the sample through the pore fluid system (see Supplementary Fig. S1 online). The two alumina (Al_2_O_3_) spacers were placed on the top and bottom of the sample (Fig. 5), allowing an even distribution of argon through the sample through a grooved cross on the face of the spacer. A type K thermocouple was inserted through the top hole of the pore fluid system and was used to monitor the temperature on top of the sample and consequently to control the furnace power. The whole assembly was inserted in a 15 mm diameter iron jacket (Fig. 5), which, having a low strength above 380 °C, ensures very good sealing around the sample to avoid any fluid bypass between the sample and jacket throughout the experiment^51,52^. Additionally, a “heat stopper” (a heat insulation tube made in-house) was placed around the jacket immediately below the upper piston to prevent overheating of the top plug (see Supplementary Figs. S1 and S2 online). For the narrow, 10 mm diameter samples, a lathe was used to fit the jacket tightly around the sample, preventing any fluid bypass between the sample and the jacket. The role of the jacket was to isolate the sample from the surrounding confining medium. A Viton O-ring sealed the gap between the jacket and the steel pistons (Fig. 5).

### Experimental approach

First, the assembly was inserted into the furnace (see Supplementary Figs. S1 and S2 online) to ensure a constant temperature along the sample, with maximum variations of 2 °C across the sample length. Then, the furnace containing the assembly was placed into the Paterson apparatus (see Supplementary Figs. S1 and S2 online)^30^. The furnace structure and insulation ensured that the target temperature was reached only in the sample, with steep temperature gradients above and below the specimen, preventing heating of the system outside the investigated rock. The confining pressure system and the pore fluid system were flushed five times with argon up to a pressure of approximately 5–8 MPa prior to application of the confining pressure. This ensured the removal of all the air and moisture initially present and guaranteed that only argon was present in the system. After flushing, the confining pressure and the pore fluid pressure were raised to near the target pressures. The confining and pore pressure were initially set slightly below the target pressures to prevent overpressurization during heating. Heating was carried out at a rate of approximately 10 °C/min. When the target experimental temperature was reached and final pressure adjustments were made, a 10-min equilibration pause was made before the start of the experiment.

We conducted deformations at higher temperatures and strain rates than those usually found in brittle–ductile condition regions to reproduce the natural conditions of brittle–ductile deformation in the laboratory, following the previous study of Violay et al.^13^. All experiments were conducted at an effective pressure of 100 MPa (150 MPa confining and 50 MPa pore fluid pressure). Nine Westerly granite samples were tested at five different temperature conditions (650 °C, 700 °C, 750 °C, 800 °C, and 850 °C) under varying strain rates (Table 2). All the experiments were run at strain rates < 3.4·10^–6^ s^−1^ (Table 2) to ensure fully drained conditions during the entire deformation and, thus, ensure correct permeability measurements. The degrees of consolidation of > 0.98 and characteristic pore pressure diffusion times of < 6.8 s were found for all the run experiments, indicating fully drained conditions throughout the deformation (with the exception of the short failure stage, where drained conditions could not be guaranteed)^53,54^.

The pore pressure oscillation method (described in detail in the Supplementary Information) records the permeability continuously without having to interrupt the ongoing specimen deformation^19^. The argon viscosity and bulk modulus necessary for the permeability computation (see Supplementary Information) were retrieved from Lemmon et al.^55^. The pump connected to the pore fluid system allowed a precise target pore-fluid pressure to be reached and therefore induced oscillations at different amplitudes and frequencies. Despite the high pressure resolution of 0.001 MPa of the pump employed, its large capacity, 70 ml, and the difficulty to accurately measure the small volume of the pore fluid pipes and connectors, did not allow an accurate pore volume change to be determined^56^; therefore, the determination of the porosity change was not possible in this study.

To perform the permeability measurements, we applied 0.005 Hz oscillations with a 2 MPa amplitude to the upstream reservoir. To ensure a clear signal suitable for data processing in the downstream reservoir (2.32·10^–6^ m^3^), the frequency and amplitude were chosen as a function of the specimen permeability and porosity. High-permeability rocks require high frequencies with low amplitudes, whereas low-porosity rocks may require low frequencies and high amplitudes. To determine the initial permeability of the rock sample, several oscillations (~ 20) were made before deformation. As soon as the starting permeability was accurately recorded, a constant power output was set to the motor to deform the rock sample at a constant rate. This procedure accurately tracks the permeability evolution during the entire deformation.

Both the upstream and downstream pressures were measured by pressure gauges, with an accuracy of < 0.125 MPa at the pump and outlet of the bottom pressure pipe, respectively (see Supplementary Fig. S1 online). The internal force and displacement were recorded, with resolutions of 0.01 kN and 0.001 mm, respectively, directly below the sample assembly, by the internal load cell (see Supplementary Figs. S1 and S2 online). The confining pressure was recorded with a resolution of 1 MPa directly at the pressure vessel, and all the recorded data were sampled with a frequency of 1 Hz throughout the experiment. Several corrections were applied to the raw data to analyze the experimental output (see the Supplementary Information for a detailed explanation of the corrections and analysis of the raw data). The data were then corrected for machine distortion, i.e., the deformation that the Paterson apparatus undergoes when deforming the sample, to allow for the correct computation of the true axial strain applied to the rock specimen (Supplementary Information; Chakrabarty^57^). Subsequently, the strain rate could be computed by correcting the internal force for the metal jacket rheology. The last correction required was the “barreling effect”, which accounts for stress variations due to changes in the sample shape during deformation. The data analysis and corrections are described in detail in the Supplementary Information.

## Supplementary Information

Supplementary Information.

## Data Availability

Datasets for this research are available in this in-text data citation reference: Petrini^58^.

## References

[1] Finger JT, Blankenship DA (2012). Handbook of Best Practices for Geothermal Drilling.

[2] Rossi, E., Kant, M., Madonna, C., Saar, M. O. & Rudolf von Rohr, P. In *SCCER-SoE Annual Conference 2017, Birmensdorf, Switzerland, September 14–15, 2017* (ed Electricity Swiss Competence Center for Energy Research–Supply of) 48 (SCCER-SoE, 2017).

[3] Elders WA, Friðleifsson GÓ, Albertsson A (2014). Drilling into magma and the implications of the Iceland Deep Drilling Project (IDDP) for high-temperature geothermal systems worldwide. Geothermics.

[4] Scott S, Driesner T, Weis P (2015). Geologic controls on supercritical geothermal resources above magmatic intrusions. Nat. Commun..

[5] Watanabe N (2017). Potentially exploitable supercritical geothermal resources in the ductile crust. Nat. Geosci..

[6] Gleeson T, Ingebritsen S (2017). Crustal Permeability.

[7] Bernabe Y, Brace WF, Evans B (1982). Permeability, porosity and pore geometry of hot-pressed calcite. Mech. Mater..

[8] Brace WF, Walsh JB, Frangos WT (1968). Permeability of granite under high pressure. J. Geophys. Res..

[9] Selvadurai APS, Głowacki A (2008). Permeability hysterisis of limestone during isotropic compression. Groundwater.

[10] Wenning QC, Madonna C, de Haller A, Burg JP (2018). Permeability and seismic velocity anisotropy across a ductile–brittle fault zone in crystalline rock. Solid Earth.

[11] Bernabe Y (1987). The effective pressure law for permeability during pore pressure and confining pressure cycling of several crystalline rocks. J. Geophys. Res. Solid Earth.

[12] Bakker RR, Violay MES, Benson PM, Vinciguerra SC (2015). Ductile flow in sub-volcanic carbonate basement as the main control for edifice stability: New experimental insights. Earth Planet. Sci. Lett..

[13] Violay M, Heap MJ, Acosta M, Madonna C (2017). Porosity evolution at the brittle-ductile transition in the continental crust: Implications for deep hydro-geothermal circulation. Sci. Rep..

[14] Violay M, Gibert B, Mainprice D, Burg JP (2015). Brittle versus ductile deformation as the main control of the deep fluid circulation in oceanic crust. Geophys. Res. Lett..

[15] Miller SA (2004). Aftershocks driven by a high-pressure CO_2_ source at depth. Nature.

[16] Majer EL (2007). Induced seismicity associated with Enhanced Geothermal Systems. Geothermics.

[17] Shmonov, V. M., Vitovtova, V. M. & Zarubina, I. V. In *Fluids in the Crust: Equilibrium and Transport Properties* (eds Shmulovich, K. I. *et al.*) 285–313 (Springer Netherlands, 1995).

[18] Zaraisky, G. P. & Balashov, V. N. In *Fluids in the Crust: Equilibrium and transport properties* (eds Shmulovich, K. I. *et al.*) 253–284 (Springer Netherlands, 1995).

[19] Mitchell, T. M. & Faulkner, D. R. Experimental measurements of permeability evolution during triaxial compression of initially intact crystalline rocks and implications for fluid flow in fault zones. J. Geophys. Res. Solid Earth 10.1029/2008JB005588 (2008).

[20] Suri P, Azeemuddin M, Zaman M, Kukreti AR, Roegiers JC (1997). Stress-dependent permeability measurement using the oscillating pulse technique. J. Petrol. Sci. Eng..

[21] De Paola N, Faulkner DR, Collettini C (2009). Brittle versus ductile deformation as the main control on the transport properties of low-porosity anhydrite rocks. J. Geophys. Res. Solid Earth.

[22] Fischer GJ, Paterson M, Evans B, Wong T-F (1992). Chapter 9 measurement of permeability and storage capacity in rocks during deformation at high temperature and pressure. International Geophysics.

[23] Zhu W, Wong T-F (1997). The transition from brittle faulting to cataclastic flow: Permeability evolution. J. Geophys. Res. Solid Earth.

[24] Zoback MD, Byerlee JD (1975). The effect of microcrack dilatancy on the permeability of westerly granite. J. Geophys. Res..

[25] Coelho G (2015). Permeability of sheeted dykes beneath oceanic ridges: Strain experiments coupled with 3D numerical modeling of the Troodos Ophiolite, Cyprus. Tectonophysics.

[26] Sander R, Pan Z, Connell LD (2017). Laboratory measurement of low permeability unconventional gas reservoir rocks: A review of experimental methods. J. Nat. Gas Sci. Eng..

[27] Noël C, Passelègue FX, Violay M (2021). Brittle faulting of ductile rock induced by pore fluid pressure build-up. J. Geophys. Res. Solid Earth.

[28] Robin P-YF (1973). Note on effective pressure. J. Geophys. Res..

[29] Jaeger JC, Cook NGW, Zimmerman R (2007). Fundamentals of Rock Mechanics.

[30] Paterson, M. A high-pressure, high-temperature apparatus for rock deformation. In *International Journal of Rock Mechanics and Mining Sciences and Geomechanics Abstracts* **7**, 517,IN513,525–524,IN514,526, 10.1016/0148-9062(70)90004-5 (1970).

[31] Heap MJ, Faulkner DR (2008). Quantifying the evolution of static elastic properties as crystalline rock approaches failure. Int. J. Rock Mech. Min. Sci..

[32] Blake OO, Faulkner DR, Tatham DJ (2019). The role of fractures, effective pressure and loading on the difference between the static and dynamic Poisson’s ratio and Young’s modulus of Westerly granite. Int. J. Rock Mech. Min. Sci..

[33] Tullis J, Yund RA (1977). Experimental deformation of dry westerly granite. J. Geophys. Res..

[34] Crawford BR (2017). Incorporating scale-dependent fracture stiffness for improved reservoir performance prediction. Rock Mech. Rock Eng..

[35] Petrini C (2020). Seismo-hydro-mechanical modelling of the seismic cycle: Methodology and implications for subduction zone seismicity. Tectonophysics.

[36] Faulkner DR, Rutter EH (2001). Can the maintenance of overpressured fluids in large strike-slip fault zones explain their apparent weakness?. Geology.

[37] Farquharson J, Heap MJ, Baud P, Reuschlé T, Varley NR (2016). Pore pressure embrittlement in a volcanic edifice. Bull. Volcanol..

[38] Watanabe N, Egawa M, Sakaguchi K, Ishibashi T, Tsuchiya N (2017). Hydraulic fracturing and permeability enhancement in granite from subcritical/brittle to supercritical/ductile conditions. Geophys. Res. Lett..

[39] Keranen KM, Weingarten M (2018). Induced seismicity. Annu. Rev. Earth Planet. Sci..

[40] Carman PC (1939). Permeability of saturated sands, soils and clays. J. Agric. Sci..

[41] Connolly JAD, Podladchikov YY (2000). Temperature-dependent viscoelastic compaction and compartmentalization in sedimentary basins. Tectonophysics.

[42] Morency C, Huismans RS, Beaumont C, Fullsack P (2007). A numerical model for coupled fluid flow and matrix deformation with applications to disequilibrium compaction and delta stability. J. Geophys. Res.-Solid Earth.

[43] Asanuma H, Muraoka H, Tsuchiya N, Ito H (2012). The concept of the Japan Beyond-Brittle Project (JBBP) to develop EGS reservoirs in ductile zones. Geotherm. Resour. Council Trans..

[44] Tsuchiya, N. *et al.* In *Proceedings World Geothermal Congress 2015.*

[45] Sibson RH (1982). Fault zone models, heat flow, and the depth distribution of earthquakes in the continental crust of the United States. Bull. Seismol. Soc. Am..

[46] Sibson RH (1977). Fault rocks and fault mechanisms. J. Geol. Soc..

[47] Moore DE, Lockner DA (1995). The role of microcracking in shear-fracture propagation in granite. J. Struct. Geol..

[48] Moore, D. E., Morrow, C. A. & Byerlee, J. D. Fluid-rock interaction and fracture development in “crystalline” rock types. Report No. 87-279, (1987).

[49] Scholz, C. *Preface: A Short Geophysical History of Westerly Granite*. (1986).

[50] Paterson M, Wong T-F (2005). Experimental Rock Deformation—The Brittle Field.

[51] Kushnir ARL, Martel C, Champallier R, Wadsworth FB (2017). Permeability evolution in variably glassy basaltic andesites measured under magmatic conditions. Geophys. Res. Lett..

[52] Frost HJ, Ashby MF (1982). Deformation-Mechanism Maps: The Plasticity and Creep of Metals and Ceramics.

[53] Gibson RE, Henkel DJ (1954). Influence of duration of tests at constant rate of strain on measured “drained” strength. Géotechnique.

[54] Montserrat S, Tamburrino A, Roche O, Niño Y (2012). Pore fluid pressure diffusion in defluidizing granular columns. J. Geophys. Res. Earth Surf..

[55] Lemmon, E. W., McLinden, M. O. & Friend, D. G. *Thermophysical Properties of Fluid Systems* http://webbook.nist.gov (2016).

[56] Fischer GJ, Evans B, Wong T-F (1992). Chapter 8 the determination of permeability and storage capacity: pore pressure oscillation method. International Geophysics.

[57] Chakrabarty J (2010). Applied Plasticity.

[58] Petrini C (2020). Data of Permeability Measurements at High Pressure and Temperature During Axial Deformation.

