# Supplementary material for: Inversion in the permeability evolution of deforming Westerly granite near the brittle–ductile transition
Source: Sci Rep. 2021 Dec 15;11:24027. doi: 10.1038/s41598-021-03435-0 (PMC8674356; doi:10.1038/s41598-021-03435-0)
Supplement: Supplementary file 1 — Supplementary Information. [file 41598_2021_3435_MOESM1_ESM.pdf]

Inversion in the permeability evolution of deforming Westerly granite near the brittle-ductile transition

Claudio Petrini[1, *, ⸸], Claudio Madonna[2, *, ⸸], and Taras Gerya[1, ⸸]

[1] Institute of Geophysics, Department of Earth Sciences, ETH Zurich, Zürich, Switzerland.

[2] Geological Institute, Department of Earth Sciences, ETH Zurich, Zürich, Switzerland.

* Corresponding authors: claudio.petrini@erdw.ethz.ch, claudio.madonna@erdw.ethz.ch

⸸ These authors contributed equally to this work.

Data correction and error propagation

Different corrections have to be applied to the raw data recorded during the triaxial experiment. Post processing is done with a MATLAB code, which reads the output files recorded by the Eurotherm iTools software (Schneider Electric) and performs all the necessary corrections on the raw data. The corrections account for:

- Apparatus distortion
- Jacket correction
- The "Barrelling effect"

The error propagation was done following equation (S1) from Taylor [59]:

$$\delta q = \sqrt{\sum_{i=1}^{n}\left(\frac{\partial q}{\partial x_i}\delta x_i\right)^2} = \sqrt{\left(\frac{\partial q}{\partial x}\delta x\right)^2 + \left(\frac{\partial q}{\partial y}\delta y\right)^2 + \ldots + \left(\frac{\partial q}{\partial z}\delta z\right)^2} \tag{S1}$$

where δq is the total error of any function q. δx, δy, …, δz are the errors of the different parameters composing equation q. $\frac{\partial q}{\partial x_i}$ represents the derivative of function q after the variable x_i. As shown in Taylor [59], the average uncertainty of a set of measurements, can be defined as its standard deviation. This error propagation formula is only valid for independent random errors.

In the following sections the different corrections and derivation of them are presented. If not stated differently, the measurement error is taken either as the uncertainty given by the manufacturer or the last significant digit of the measurement apparatus.

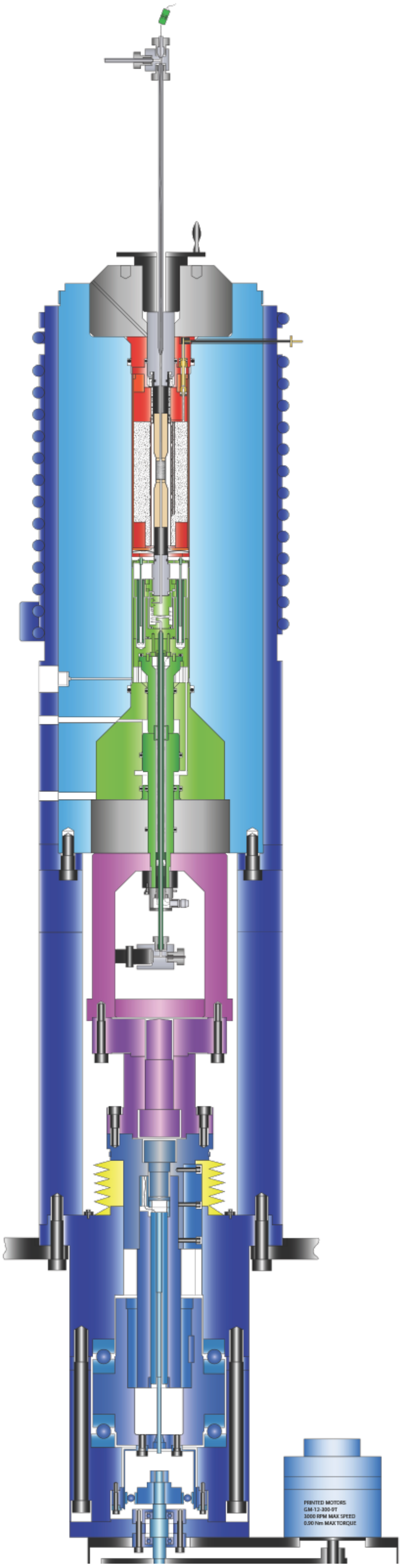

Supplementary Figure S1: Paterson apparatus #6. Drawing after Paterson Instruments PTY LTD [30,60]. The blue parts represent the pressure vessel, the machine structure and the motor controlling the piston, violet parts are the external load cell, green components are the internal load cell, red part is the furnace with inside the sample assembly. The grey represents the top and bottom pistons, as well as the pore fluid pipes and the top and bottom closing caps. Drawing not to scale.

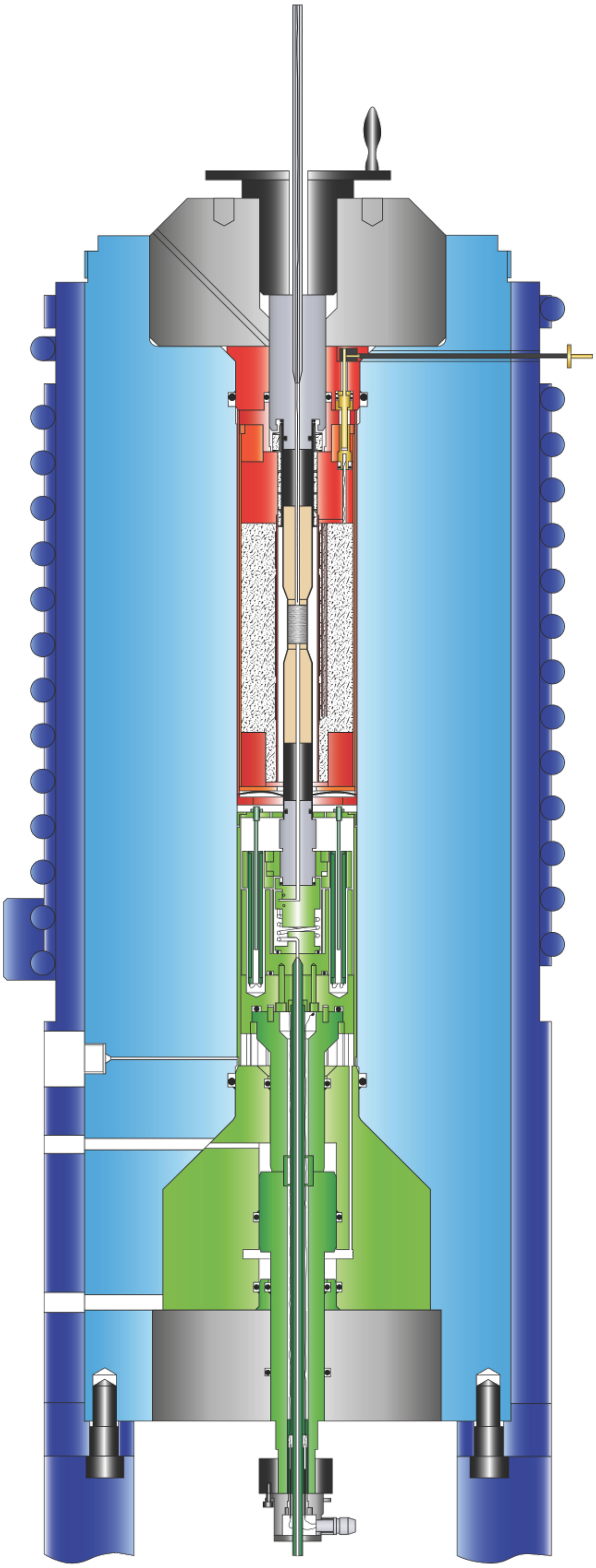

Supplementary Figure S2: Upper part of the Paterson apparatus #6. Drawing after Paterson Instruments PTY LTD [30,60]. Blue is the pressure vessel. Green is the internal load cell. Red is the furnace with inside the sample assembly. Grey: the top and bottom caps of the triaxial gas apparatus. Drawing not to scale.

Apparatus distortion

To retrieve the strain of the deforming sample from the measured data, the data must be corrected for the apparatus distortion due to the high stresses which the entire machine is subjected to. Under high confining pressures and stresses the entire apparatus deforms together with the sample and the measured data include both deformations, which must be separated. The stiffness of the load cell and the machine increases with increasing confining pressure and must be determined for each condition and machine separately. First the compression of apparatus, at a given confining pressure, is calculated:

$$\beta_{LC} = \frac{IF}{k} \tag{S2}$$

with IF the measured internal force and k the stiffness of the machine at the given confining pressure. Once the distortion of the Paterson apparatus is known, the actual displacement of the sample during the deformation is determined as follows:

$$dx_{sample} = dx_{sampleold} - \beta_{LC} \tag{S3}$$

with $dx_{sampleold}$ being the recorded displacement of the sample. At this stage the effective strain of the sample is easily computed:

$$\varepsilon_{sample} = \frac{dx_{sample}}{l_{sample}} \tag{S4}$$

where l_{sample} is the initial length of the rock sample.
Once the effective strain is computed, in order to account for the instant change in length during deformation, the true strain (in the following subchapters referred as ε_a) has to be computed by integrating over the total length change the evolving sample length L, with dL being the incremental change in sample length [57]:

$$\begin{aligned}\varepsilon_{true} &= \int_{l}^{l_{sample}} \frac{dL}{L} = \ln(L)\Big|_{l}^{l_{sample}} = \ln(l_{sample}) - \ln(l) = \ln\left(\frac{l_{sample}}{l}\right) \\ &= \ln\left(\frac{l_{sample}}{(l_{sample} - dx_{sample})}\right) = \ln\left(\frac{1}{(1-\varepsilon_{sample})}\right) = -\ln(1-\varepsilon_{sample})\end{aligned} \tag{S5}$$

Beside the computation of the true strain, errors produced during the different measures and computations propagate and must be included in the resulting strain error. The error is computed as follow by using the equation (S1) assuming no error for the stiffness of the apparatus (not available) [59]:

$$\delta\varepsilon_{sample} = \sqrt{\left(\frac{\partial\varepsilon_{sample}}{\partial dx_{sampleold}}\delta dx_{sampleold}\right)^2 + \left(\frac{\partial\varepsilon_{sample}}{\partial IF}\delta IF\right)^2 + \left(\frac{\partial\varepsilon_{sample}}{\partial k}\delta k\right)^2 + \left(\frac{\partial\varepsilon_{sample}}{\partial l_{sample}}\delta l_{sample}\right)^2} \tag{S6}$$

$$\delta\varepsilon_{true} = \sqrt{\left(\frac{\partial\varepsilon_{true}}{\partial\varepsilon_{sample}}\delta\varepsilon_{sample}\right)^2} \tag{S7}$$

which becomes:

$$\delta\varepsilon_{sample} = \sqrt{\left(\frac{1}{l_{sample}}\delta dx_{sampleold}\right)^2 + \left(-\frac{1}{kl_{sample}}\delta IF\right)^2 + \left(\frac{IF}{k^2 l_{sample}}\delta k\right)^2 + \left(\left(-\frac{dx_{sampleold}}{l_{sample}^2} + \frac{IF}{kl_{sample}^2}\right)\delta l_{sample}\right)^2} \tag{S8}$$

$$\delta\varepsilon_{true} = \sqrt{\left(\frac{1}{\left(1-\varepsilon_{sample}\right)}\delta\varepsilon_{sample}\right)^2} \tag{S9}$$

Strain rate computation

The strain rate is defined as the change of strain in time and can, therefore, be expressed as the time derivative of the strain:

$$\dot{\varepsilon} = \frac{\partial\varepsilon}{\partial t} \tag{S10}$$

As every digital recorded signal, the data retrieved from the experiment are all in form of discrete time series; this means that only an approximation of the strain rate at a given time can be computed by discretising the time derivative of the strain:

$$\dot{\varepsilon} = \frac{\Delta\varepsilon}{\Delta t} = \frac{\varepsilon_{t+\Delta t} - \varepsilon_t}{\Delta t} \tag{S11}$$

A good approximation of the strain rate is computed using the discretisation method. The strain rate is needed as input to compute the force involved in the deformation of the specimen jacket by means of the power-law creep [52]. Due to the very small strain acting before and after failure of the rock sample, the computed strain rate presents significant noise that should be filtered out by a Low-pass filter.

For simplification, the error on the recorded time is neglected. The error related to the strain rate is computed as follows [59]:

$$\delta\dot{\varepsilon} = \sqrt{\left(\frac{\partial\dot{\varepsilon}}{\partial\varepsilon_{t+\Delta t}}\delta\varepsilon_{t+\Delta t}\right)^2 + \left(\frac{\partial\dot{\varepsilon}}{\partial\varepsilon_t}\delta\varepsilon_t\right)^2} = \sqrt{\left(\frac{1}{\Delta t}\delta\varepsilon_{t+\Delta t}\right)^2 + \left(-\frac{1}{\Delta t}\delta\varepsilon_t\right)^2} \tag{S12}$$

For consistency with the filtered data, the error computed is also filtered in the same way as the strain rate.

Jacket rheology correction

During the sample deformation, a given amount of deformation goes into the jacket, which isolate the sample from the confining medium. To discriminate between the amount of deformation belonging to the jacket and the actual deformation of the rock sample, the measured applied force must be corrected for the rheology of the jacket. The force absorbed by the jacket depends on the jacket composition and strongly on temperature. At low temperatures, the correction is important, since the jacket takes over a significant amount of force giving a distorted rheology of the rock, whereas at high temperatures, the jacket contribution becomes negligible. The jacket must be chosen so that it is not too week to fail during the experiment and not too strong to influence the deformation of the specimen, for this reason up to 600 °C, copper (Cu) jackets are used, whereas above this temperature, iron (Fe) jackets are applied.

Either copper or iron tubes with an inner diameter of 15.10 ± 0.01 mm and an outer diameter of 15.50 ± 0.01 were used in the experiment. These tubes are assumed to be pure in composition, allowing the application of the rate equation for power-law creep and the flaw parameters presented in Frost and Ashby [52]. The overall deformation of the metal is given by different deformation mechanism, which becomes the major deformation mechanism at given pressure and temperature, as shown in Covey-Crump et al. [61].

The plastic flow of solid materials (e.g., metals) is driven by the deviatoric component of the stress field (shear stress) [52]. Shear stress and shear strain rate can be related to von Mises equivalent stress and equivalent strain rate as follows:

$$\tau_j = \frac{\sigma_{eq}}{\sqrt{3}} \tag{S13}$$

$$\dot{\gamma} = \sqrt{3}\dot{\varepsilon}_{eq} \tag{S14}$$

In case of pure axial deformation (pure shear), the equivalent stress and strain rate are equal to the axial stress and strain rate, therefore shear stress and shear strain rate can be easily rewritten in the following form:

$$\tau_j = \frac{\sigma_{ax}}{\sqrt{3}} \tag{S15}$$

$$\dot{\gamma} = \sqrt{3}\dot{\varepsilon}_{ax} \tag{S16}$$

This formulation allows us to compute the amount of axial stress (later denoted as σ_{jacket}) absorbed by the deformation of the jacket using the power-law creep formulated by Frost and Ashby [52].

The power-law creep, which can be used for the jacket correction either in pure shear experiments (axial deformation) or in simple shear experiments (torsion experiments), includes different deformation mechanisms such as core and lattice diffusion and relates shear strain rate $\dot{\gamma}$ and shear stress τ_j as follows:

$$\dot{\gamma} = \frac{A_s D_{eff} G b}{kT}\left(\frac{\tau_j}{G}\right)^n \tag{S17}$$

where A_s is the Dorn constant relating shear stress and strain rate, b the Burgers vector, τ_j the shear stress acting on the metal jacket, G the shear modulus, k the Boltzmann's constant, T the temperature, and n a material exponent. D_{eff} is the effective diffusion coefficient:

$$D_{eff} = D_v\left[1+\frac{10a_cD_c}{b^2D_v}\left(\frac{\tau_j}{G}\right)^2\right] \tag{S18}$$

with D_v and a_cD_c the diffusion coefficients for lattice diffusion and core diffusion:

$$D_v = D_{0v}e^{\left(-\frac{Q_v}{RT}\right)} \tag{S19}$$

$$a_cD_c = a_cD_{0c}e^{\left(-\frac{Q_c}{RT}\right)} \tag{S20}$$

where R is the universal gas constant, Q_v and Q_c the activation energy for lattice diffusion and core diffusion respectively, D_{0v} and a_cD_{0c} the pre-exponential term for lattice diffusion and core diffusion.

In the case of iron jackets, the diffusion coefficient D_v for lattice diffusion is taken to be a combination between paramagnetic and ferromagnetic α-iron diffusion coefficients [52,62]:

$$D_v = fD_{v-para} + \left(1-f\right)D_{v-ferro} \tag{S21}$$

with f a temperature dependent partition function between ferromagnetic and paramagnetic iron:

$$f = \frac{1}{2} + \frac{0.5(1-1043)}{\left(\left|(T-1043)\right|+20\right)} \tag{S22}$$

and where

$$D_{v-ferro} = D_{0v-ferro}e^{\left(-\frac{Q_{v-ferro}}{RT}\right)} \tag{S23}$$

$$D_{v-para} = D_{0v-para}e^{\left(-\frac{Q_{v-para}}{RT}\right)} \tag{S24}$$

The values of the different parameters are material dependent and are listed in Supplementary Table S1 [52].

Parameter	Copper	Iron	Units
k	$1.38064 \cdot 10^{-23}$	$1.38064 \cdot 10^{-23}$	$m^2 \cdot Kg \cdot s^{-2} \cdot K^{-1}$
b	$2.56 \cdot 10^{-10}$	$2.48 \cdot 10^{-10}$	m
R	8.31415	8.31415	$J \cdot mol^{-1} \cdot K^{-1}$
n	4.8	6.9	–
A	$7.4 \cdot 10^{5}$	$7.0 \cdot 10^{13}$	–
A_s	$(\sqrt{3})^{n+1} \cdot A$	$(\sqrt{3})^{n+1} \cdot A$	–
D_{0v}	$2.0 \cdot 10^{-5}$	(ferro) $2.0 \cdot 10^{-4}$<br>(para) $1.9 \cdot 10^{-4}$	$m^2 \cdot s^{-1}$
Q_v	197000	(ferro) 251000<br>(para) 239000	$J \cdot mol^{-1}$
$a_c D_{0c}$	$1.0 \cdot 10^{-24}$	$1.0 \cdot 10^{-23}$	$m^4 \cdot s^{-1}$
Q_c	117000	174000	$J \cdot mol^{-1}$
T_M	1356	1810	K
G_0	$4.21 \cdot 10^{4}$	(ferro) $6.4 \cdot 10^{4}$<br>(para) $6.92 \cdot 10^{4}$	MPa
ΔG	-0.54	(ferro) -0.81<br>(para) -1.31	–

Supplementary Table S1: Flaw law parameters for copper and Iron [52].

By inserting equation (S18) into equation (S17) and by rearranging the terms, a function dependent on the shear stress and that can be solved iteratively is derived:

$$\dot{\gamma} = \frac{A_s G b \tau_j^{\ n}}{kTG^n}\left(D_v + \frac{10 a_c D_c \tau_j^{\ 2}}{b^2 G^2}\right)$$

$$\dot{\gamma} = \frac{A_s G b D_v}{kTG^n}\tau_j^{\ n} + \frac{A_s G b 10 a_c D_c}{kTG^n b^2 G^2}\tau_j^{\ n+2}$$

$$\dot{\gamma} = \frac{A_s b D_v}{kTG^{n-1}}\tau_j^{\ n} + \frac{A_s 10 a_c D_c}{kTG^{n+1} b}\tau_j^{\ n+2}$$

$$f(\tau) = \frac{A_s b D_v}{kTG^{n-1}}\tau_j^{\ n} + \frac{A_s 10 a_c D_c}{kTG^{n+1} b}\tau_j^{\ n+2} - \dot{\gamma} = 0$$

$$f(\tau) = M\tau_j^{\ n} + N\tau_j^{\ n+2} - \dot{\gamma} = 0 \qquad \text{(S25)}$$

with *M* and *N* being two constants:

$$M = \frac{A_s b D_v}{kTG^{n-1}} \qquad \text{(S26)}$$

$$N = \frac{A_s 10 a_c D_c}{kTG^{n+1} b} \qquad \text{(S27)}$$

A good way to solve the power-law creep for the shear stress (eq. S17) is to use an iterative approach. To have a high accuracy convergence the bisection method is used to solve for the shear stress coefficient, similarly as proposed in Covey-Crump et al. [61]. This slow iterative method insures a stable and accurate solution.
The shear modulus is temperature dependent and for copper can be described as follows [52]:

$$G = G_0\left(1 + \frac{T - 300}{T_M}\right)\Delta G \qquad \text{(S28)}$$

with T_M the melting temperature, G_0 the shear modulus at 300 °C and ΔG the temperature dependence of the shear modulus:

$$\frac{T_M}{G_0}\frac{dG}{dT} \tag{S29}$$

For the shear modulus of iron, the dependency of temperature gets more complex at temperatures lower than the transition from ferromagnetic to paramagnetic (Curie temperature, T_c, 1043 °K = 770 °C), behaving non-linearly [52]:

if 300 °C < T ≤ 650 °C $$G = G_{0-ferro}\left(1+\frac{T-300}{T_M}\Delta G_{ferro}\right) - K_1\left(T-573\right)^2 \tag{S30}$$

if 650 °C < T ≤ 770 °C $$G = G_{0-ferro}\left(1+\frac{T-300}{T_M}\Delta G_{ferro}\right) - K_1\left(T-573\right)^2 - K_2\left(T-923\right)^2 \tag{S31}$$

if T > 770 °C $$G = G_{0-para}\left(1+\frac{T-300}{T_M}\Delta G_{para}\right) \tag{S32}$$

where K_1 and K_2 are constants equal to $3.2\cdot10^{-2}$ MPa/K^2 and $2.4\cdot10^{-2}$ MPa/K^2 respectively and $G_{0-ferro}$, ΔG_{ferro} and G_{0-para}, ΔG_{para}, are the shear modulus at 300 °C and change in shear modulus for ferromagnetic and paramagnetic iron respectively. Below 300 °C the behaviour is not defined by the authors [52]; therefore, we assume the shear modulus to be equal to the shear modulus at 300 °C.

To be able to compute the amount of deformation, which goes into the jacket, the area of the jacket cross-section must be computed:

$$A_{jinit} = \frac{(d_{ext}^{\;2} - d_{in}^{\;2})}{4}\pi \tag{S33}$$

with $d_{ext/in}$ the external and internal diameter of the metal tube used for the jacket.

Since the jacket deforms together with the rock, a correction for a barrelling effect (cf. next section) of the jacket has also to be performed and is non-negligible at high experimental temperature, due to the high ductility of the metal at such conditions. The procedure is similar to the one used for the rock specimen (cf. next section); however, the jacket is assumed to have no grain compressibility and a negligible pore space change. For these reasons, the change in area is assumed to happen at constant jacket volume and change only in relation to axial strain ε_a:

$$lA_{jacket} = l_{init}A_{jinit} = V_{jacket} \tag{S34}$$

with, V_{jacket} the volume of the jacket, A_{jacket} and A_{jinit} the cross-sectional area of the corrected and initial jacket and l and l_{init} the length of the deformed and un-deformed jacket. By substituting in equation (S34) $l = l_{init}\left(1-\varepsilon_a\right)$ and rearranging the terms, the resulting corrected jacket area is as follows:

$$A_{jacket} = A_{jinit}\left(1-\varepsilon_a\right) \tag{S35}$$

To retrieve the force needed to deform the metal jacket with the measured strain rate, the computed stress is multiplied with the corrected cross-sectional area of the tube:

$$IF_{jacket} = \sigma_{jacket} A_{jacket} \tag{S36}$$

To obtain the force acting on the rock specimen the force absorbed by the jacket deformation is subtracted from the total force applied during the deformation:

$$IF_{spec} = IF_{tot} - IF_{jacket} \tag{S37}$$

Although the data used to produce the deformation maps and derive the flow law equations and parameters are experimentally defined and contain a given amount of error. Here, for simplification, we consider the parameters given in Frost and Ashby [52] as precise values without error and the equations proposed as the correct description for the deformation of interest. The principal mechanism during deformation of the different materials and elements is still far to be known exactly and much work is still necessary in this field of research. Furthermore, errors in equations and deformation maps are difficult to quantify and the parameters given in Frost and Ashby [52] have not a quantified error that can be used for a proper error propagation. As proposed by Frost and Ashby [52], the derived equation, the proposed parameters and the deformation maps, are an approximation of very complex and still not fully understood physical mechanisms. Therefore, unquantified uncertainties in the results must be expected. However, a rough estimate of the error in the force absorbed by the jacket is possible, even with some constant assumed to be without error, by considering the known errors, such as the error in temperature or in strain rate. The error analysis presented for the correction of the jacket rheology follows Taylor [59].
The final force error on the sample is computed as follows:

$$\delta IF_{spec} = \sqrt{\left(\delta IF_{tot}\right)^2 + \left(\delta IF_{jacket}\right)^2} \tag{S38}$$

where δIF_{tot} is given by the sensor accuracy of the Paterson apparatus, whereas δIF_{jacket} is equal to:

$$\delta IF_{jacket} = \sqrt{\left(\frac{\partial IF_{jacket}}{\partial \sigma_{jacket}} \delta\sigma_{jacket}\right)^2 + \left(\frac{\partial IF_{jacket}}{\partial A_{jacket}} \delta A_{jacket}\right)^2} = \sqrt{\left(A_{jacket}\delta\sigma_{jacket}\right)^2 + \left(\sigma_{jacket}\delta A_{jacket}\right)^2} \tag{S39}$$

The error given by the cross-sectional area of the jacket is:

$$\delta A_{jacket} = \sqrt{\left(\frac{\partial A_{jacket}}{\partial A_{jinit}} \delta A_{jinit}\right)^2 + \left(\frac{\partial A_{jacket}}{\partial \varepsilon_a} \delta\varepsilon_a\right)^2} = \sqrt{\left(\frac{1}{1-\varepsilon_a} \delta A_{jinit}\right)^2 + \left(\frac{1}{\left(1-\varepsilon_a\right)^2} \delta\varepsilon\right)^2} \tag{S40}$$

where ε_a is the axial strain and $\delta\varepsilon_a$ its error, whereas δA_{jinit} the error of the initial cross-sectional area of the jacket:

$$\delta A_{jinit} = \sqrt{\left(\frac{\partial A_{jinit}}{\partial d_{ext}}\delta d_{ext}\right)^2 + \left(\frac{\partial A_{jinit}}{\partial d_{\text{int}}}\delta d_{\text{int}}\right)^2} = \sqrt{\left(\frac{d_{ext}\pi}{2}\delta d_{ext}\right)^2 + \left(\frac{d_{in}\pi}{2}\delta d_{\text{int}}\right)^2} \qquad \text{(S41)}$$

with $\delta d_{ext/int}$ the errors of the external and internal diameter. On the other hand, even if the procedure is always the same, the computation of the error of the stress absorbed by the jacket becomes computationally slightly more intensive and similarly when solving the power-law creep for shear stress, also the computation of its error will need to be solved using the same bisection iterative approach:

$$\delta\tau_j = \sqrt{\left(\frac{\partial\tau_j}{\partial T}\delta T\right)^2 + \left(\frac{\partial\tau_j}{\partial G}\delta G\right)^2 + \left(\frac{\partial\tau_j}{\partial\dot{\gamma}}\delta\dot{\gamma}\right)^2 + \left(\frac{\partial\tau_j}{\partial D_v}\delta D_v\right)^2 + \left(\frac{\partial\tau_j}{\partial a_c D_c}\delta a_c D_c\right)^2 + \left(\frac{\partial\tau_j}{\partial\tau_j}\delta\tau_j\right)^2} \qquad \text{(S42)}$$

The different derivatives do not need to be solved iteratively, since they are not depending on the shear stress error, but only on already computed values. In order to get an equation for the shear stress, equations (S17) and (S18) need to be combined again and by simple algebra and terms rearrangement, an equation for the shear stress is derived:

$$\dot{\gamma} = \frac{A_s G b}{kT} D_v \left[1 + \frac{10 a_c D_c}{b^2 D_v}\left(\frac{\tau_j}{G}\right)^2\right]\left(\frac{\tau_j}{G}\right)^n$$

$$\dot{\gamma} = \frac{A_s G b}{kT}\left(\frac{D_v G^2 b^2 + 10 a_c D_c \tau_j^{\,2}}{G^2 b^2}\right)\left(\frac{\tau_j}{G}\right)^n$$

$$\dot{\gamma} = \frac{A_s G b}{kT}\left(\frac{D_v G^2 b^2 + 10 a_c D_c \tau_j^{\,2}}{G^{(2+n)} b^2}\right)\tau_j^{\,n}$$

$$\tau_j^{\,n} = \frac{\dot{\gamma} kT}{A_s G b}\left(\frac{G^{(2+n)} b^2}{D_v G^2 b^2 + 10 a_c D_c \tau_j^{\,2}}\right) = \frac{\dot{\gamma} kTbG^{(1+n)}}{A_s D_v G^2 b^2 + A_s 10 a_c D_c \tau_j^{\,2}}$$

$$n\ln\left(\tau_j\right) = \ln\left(\frac{\dot{\gamma} kTbG^{(1+n)}}{A_s D_v G^2 b^2 + A_s 10 a_c D_c \tau_j^{\,2}}\right)$$

$$\tau_j = \exp\left(\frac{\ln\left(\frac{\dot{\gamma} kTbG^{(1+n)}}{A_s D_v G^2 b^2 + A_s 10 a_c D_c \tau_j^{\,2}}\right)}{n}\right) = \left(\frac{\dot{\gamma} kTbG^{(1+n)}}{A_s D_v G^2 b^2 + A_s 10 a_c D_c \tau_j^{\,2}}\right)^{\frac{1}{n}} \qquad \text{(S43)}$$

The partial derivatives of equation (S43) and used to compute the error in shear stress (eq. S42) are explicitly derived as follow:

$$\frac{\partial\tau_j}{\partial T}=\frac{1}{n}\left(\frac{\dot{\gamma}kTbG^{(1+n)}}{A_sD_vG^2b^2+A_s10a_cD_c\tau_j{}^2}\right)^{\frac{1}{n}-1}\left(\frac{\dot{\gamma}kbG^{(1+n)}}{A_sD_vG^2b^2+A_s10a_cD_c\tau_j{}^2}\right)\frac{T}{T}$$

$$=\frac{1}{nT}\left(\frac{\dot{\gamma}kTbG^{(1+n)}}{A_sD_vG^2b^2+A_s10a_cD_c\tau_j{}^2}\right)^{\frac{1}{n}} \qquad \text{(S44)}$$

$$\frac{\partial\tau_j}{\partial G}=\frac{1}{n}\left(\frac{\dot{\gamma}kTbG^{(1+n)}}{A_sD_vG^2b^2+A_s10a_cD_c\tau_j{}^2}\right)^{\frac{1}{n}-1}\left[\frac{\dot{\gamma}kTb(1+n)G^n\left(A_sD_vG^2b^2+A_s10a_cD_c\tau_j{}^2\right)-\dot{\gamma}kTbG^{(1+n)}2A_sD_vGb^2}{\left(A_sD_vG^2b^2+A_s10a_cD_c\tau_j{}^2\right)^2}\right]$$

$$=\frac{1}{n}\left(\frac{\dot{\gamma}kTbG^{(1+n)}}{A_sD_vG^2b^2+A_s10a_cD_c\tau_j{}^2}\right)^{\frac{1}{n}-1}\left[\frac{\dot{\gamma}kTb(1+n)G^{(n+1)}A_sD_vGb^2+\dot{\gamma}kTb(1+n)G^nA_s10a_cD_c\tau_j{}^2-\dot{\gamma}kTbG^{(1+n)}2A_sD_vGb^2}{\left(A_sD_vG^2b^2+A_s10a_cD_c\tau_j{}^2\right)^2}\right]$$

$$=\frac{1}{n}\left(\frac{\dot{\gamma}kTbG^{(1+n)}}{A_sD_vG^2b^2+A_s10a_cD_c\tau_j{}^2}\right)^{\frac{1}{n}-1}\left[\frac{\dot{\gamma}kTbG^{(1+n)}}{A_sD_vG^2b^2+A_s10a_cD_c\tau_j{}^2}\frac{(1+n)A_sD_vGb^2+(1+n)A_sG^{-1}10a_cD_c\tau_j{}^2-2A_sD_vGb^2}{A_sD_vG^2b^2+A_s10a_cD_c\tau_j{}^2}\right]$$

$$=\frac{1}{n}\left(\frac{\dot{\gamma}kTbG^{(1+n)}}{A_sD_vG^2b^2+A_s10a_cD_c\tau_j{}^2}\right)^{\frac{1}{n}}\left[\frac{(n-1)D_vGb^2+(1+n)G^{-1}10a_cD_c\tau_j{}^2}{D_vG^2b^2+10a_cD_c\tau_j{}^2}\right]\frac{G}{G}$$

$$=\frac{(n-1)D_vG^2b^2+(1+n)10a_cD_c\tau_j{}^2}{nG\left(D_vG^2b^2+10a_cD_c\tau_j{}^2\right)}\left(\frac{\dot{\gamma}kTbG^{(1+n)}}{A_sD_vG^2b^2+A_s10a_cD_c\tau_j{}^2}\right)^{\frac{1}{n}} \qquad \text{(S45)}$$

$$\frac{\partial\tau_j}{\partial\dot{\gamma}}=\frac{1}{n}\left(\frac{\dot{\gamma}kTbG^{(1+n)}}{A_sD_vG^2b^2+A_s10a_cD_c\tau_j{}^2}\right)^{\frac{1}{n}-1}\left(\frac{kTbG^{(1+n)}}{A_sD_vG^2b^2+A_s10a_cD_c\tau_j{}^2}\right)\frac{\dot{\gamma}}{\dot{\gamma}}$$

$$=\frac{1}{n\dot{\gamma}}\left(\frac{\dot{\gamma}kTbG^{(1+n)}}{A_sD_vG^2b^2+A_s10a_cD_c\tau_j{}^2}\right)^{\frac{1}{n}} \qquad \text{(S46)}$$

$$\frac{\partial\tau_j}{\partial D_v}=-\frac{1}{n}\left(\frac{\dot{\gamma}kTbG^{(1+n)}}{A_sD_vG^2b^2+A_s10a_cD_c\tau_j{}^2}\right)^{\frac{1}{n}-1}\left(\frac{\dot{\gamma}kTbG^{(1+n)}}{\left(A_sD_vG^2b^2+A_s10a_cD_c\tau_j{}^2\right)^2}\right)A_sG^2b^2$$

$$=-\frac{G^2b^2}{n\left(D_vG^2b^2+10a_cD_c\tau_j{}^2\right)}\left(\frac{\dot{\gamma}kTbG^{(1+n)}}{A_sD_vG^2b^2+A_s10a_cD_c\tau_j{}^2}\right)^{\frac{1}{n}} \qquad \text{(S47)}$$

$$\frac{\partial\tau_j}{\partial a_cD_c}=-\frac{1}{n}\left(\frac{\dot{\gamma}kTbG^{(1+n)}}{A_sD_vG^2b^2+A_s10a_cD_c\tau_j{}^2}\right)^{\frac{1}{n}-1}\left(\frac{\dot{\gamma}kTbG^{(1+n)}}{\left(A_sD_vG^2b^2+A_s10a_cD_c\tau_j{}^2\right)^2}\right)A_s10\tau_j{}^2$$

$$=-\frac{10\tau_j{}^2}{n\left(D_vG^2b^2+10a_cD_c\tau_j{}^2\right)}\left(\frac{\dot{\gamma}kTbG^{(1+n)}}{A_sD_vG^2b^2+A_s10a_cD_c\tau_j{}^2}\right)^{\frac{1}{n}} \qquad \text{(S48)}$$

$$\frac{\partial\tau_j}{\partial\tau_j}=-\frac{1}{n}\left(\frac{\dot{\gamma}kTbG^{(1+n)}}{A_sD_vG^2b^2+A_s10a_cD_c\tau_j{}^2}\right)^{\frac{1}{n}-1}\left(\frac{\dot{\gamma}kTbG^{(1+n)}}{\left(A_sD_vG^2b^2+A_s10a_cD_c\tau_j{}^2\right)^2}\right)20a_cD_c\tau_j$$

$$=-\frac{20a_cD_c\tau_j}{n\left(D_vG^2b^2+10a_cD_c\tau_j{}^2\right)}\left(\frac{\dot{\gamma}kTbG^{(1+n)}}{A_sD_vG^2b^2+A_s10a_cD_c\tau_j{}^2}\right)^{\frac{1}{n}} \qquad \text{(S49)}$$

In a similar way to the computation of the axial stress, the error in shear stress has to be transformed using the von Mises equivalent stress relationship in the error in axial stress, so that it can be related to the computed axial stress.
The error of temperature (δT) is given by the thermocouple accuracy, whereas the error of the strain rate ($\delta\dot{\varepsilon}$) is already computed when computing the strain rate. The other errors are computed further with same procedure used until this point:

$$\delta a_c D_c = \sqrt{\left(\frac{\partial a_c D_c}{\partial T}\delta T\right)^2} = \sqrt{\left(\frac{a_c D_{0c} Q_c}{RT^2} e^{\left(-\frac{Q_c}{RT}\right)}\delta T\right)^2} \tag{S50}$$

$$\delta D_v = \sqrt{\left(\frac{\partial D_v}{\partial T}\delta T\right)^2} = \sqrt{\left(\frac{D_{0v} Q_v}{RT^2} e^{\left(-\frac{Q_v}{RT}\right)}\delta T\right)^2} \tag{S51}$$

In case of iron, the computation for the error of the pre-exponential for lattice diffusion, δD_v, must be computed considering for ferromagnetic and paramagnetic α-iron:

$$\delta D_v = \sqrt{\left(\frac{\partial D_v}{\partial D_{v-ferro}}\delta D_{v-ferro}\right)^2 + \left(\frac{\partial D_v}{\partial D_{v-para}}\delta D_{v-para}\right)^2 + \left(\frac{\partial D_v}{\partial f}\delta f\right)^2} \tag{S52}$$

becoming:

$$\delta D_v = \sqrt{\left((1-f)\delta D_{v-ferro}\right)^2 + \left(f\delta D_{v-para}\right)^2 + \left(\left(D_{v-para} - D_{v-ferro}\right)\delta f\right)^2} \tag{S53}$$

with

$$\delta D_{v-ferro} = \sqrt{\left(\frac{\partial D_{v-ferro}}{\partial T}\delta T\right)^2} = \sqrt{\left(\frac{D_{0v-ferro} Q_{v-ferro}}{RT^2} e^{\left(-\frac{Q_{v-ferro}}{RT}\right)}\delta T\right)^2} \tag{S54}$$

$$\delta D_{v-para} = \sqrt{\left(\frac{\partial D_{v-para}}{\partial T}\delta T\right)^2} = \sqrt{\left(\frac{D_{0v-para} Q_{v-para}}{RT^2} e^{\left(-\frac{Q_{v-para}}{RT}\right)}\delta T\right)^2} \tag{S55}$$

$$\delta f = \sqrt{\left(\frac{\partial f}{\partial T}\delta T\right)^2} = \sqrt{\left(-\frac{0.5(1-1043)}{\left(|(T-1043)|+20\right)}\frac{(T-1043)}{|T-1043|}\delta T\right)^2} \tag{S56}$$

The last error to compute is the error produced when computing the shear modulus, which in case of copper is very simple and straight forward:

$$\delta G = \sqrt{\left(\frac{\partial G}{\partial T}\delta T\right)^2} = \sqrt{\left(\frac{G_0 \Delta G}{T_M}\delta T\right)^2} \tag{S57}$$

whereas when dealing with iron, the non-linearity below the Curie temperature must be considered and the error for the shear modulus of iron becomes:

if 300 °C < T ≤ 650 °C $$\delta G=\sqrt{\left(\frac{\partial G}{\partial T}\delta T\right)^2}=\sqrt{\left(\left(\frac{G_{0-ferro}\Delta G_{ferro}}{T_M}+2K_1(T-573)\right)\delta T\right)^2} \quad \text{(S58)}$$

if 650 °C < T ≤ 770 °C $$\delta G=\sqrt{\left(\frac{\partial G}{\partial T}\delta T\right)^2}=\sqrt{\left(\left(\frac{G_{0-ferro}\Delta G_{ferro}}{T_M}+2K_1(T-573)+2K_2(T-923)\right)\delta T\right)^2} \quad \text{(S59)}$$

if T > 770 °C $$\delta G=\sqrt{\left(\frac{\partial G}{\partial T}\delta T\right)^2}=\sqrt{\left(\frac{G_{0-para}\Delta G_{para}}{T_M}\delta T\right)^2} \quad \text{(S60)}$$

Barrelling effect correction

The "Barrelling effect correction" compensates the change in shape of the sample during the deformation. During the deformation process, the sample deforms assuming a shape close to a barrel, with consequently an increase of the cross-sectional area. This shape is given from the fact that, due to piston friction, the top and bottom area of the sample do not deform, whereas the internal sample does, forming a wine barrel like shape. The need for this correction is mostly needed in the brittle-ductile regime, where this effect is mostly present [50]. In the brittle regime, particularly in confined triaxial experiments , however, it seems that this effect is negligible [50].

To be able to compute correctly the applied stress, the so-called true stress, on the sample, this correction for the increasing cross-sectional area must be applied. Starting from the volume computation, is possible to derive the formula to compute the new deformed cross-sectional area:

$$lA_{samplecorr}=V \quad \text{(S61)}$$

with l the deformed length of the sample, $A_{samplecorr}$ the cross-sectional area of the rock sample, assumed to be a circle, V the volume of the deformed sample. The deformed length of the sample, as well as the volume, can both be described by means of the axial and volumetric strain, ε_a and ε_v respectively:

$$l=l_{init}\left(1-\varepsilon_a\right) \quad \text{(S62)}$$

$$V=V_{init}\left(1-\varepsilon_v\right) \quad \text{(S63)}$$

with l_{init} and V_{init} the initial length and volume of the rock specimen. The initial volume can be easily computed by the initial length and area of the sample:

$$V_{init}=A_{sample}l_{init} \quad \text{(S64)}$$

By using the above expression, the volume can be rewritten as:

$$l_{init}\left(1-\varepsilon_a\right)A_{samplecorr}=A_{sample}l_{init}\left(1-\varepsilon_v\right) \quad \text{(S65)}$$

and after some simplification and repositioning of the terms, the formula for computing the corrected cross-sectional area of the rock specimen becomes [63,64]:

$$A_{samplecorr} = A_{sample} \frac{(1-\varepsilon_v)}{(1-\varepsilon_a)} \tag{S66}$$

This formula assumes a cylindrical deformation of the sample (constant cross-sectional area throughout the entire sample height). More precise and complex formulations for barrel-shape deformation can be found in the literature (e.g., Omar and Sadrekarimi [64]), however they differ only by 2% or 3% and the assumption taken to derive this formulas are not necessarily more accurate than the one proposed here [63]. In case the volumetric strain of the deformed sample cannot be assessed, ε_v can be assumed to be equal zero and equation (S66) can be rewritten as follow:

$$A_{samplecorr} = A_{sample} \frac{1}{(1-\varepsilon_a)} \tag{S67}$$

After successfully corrected the area, the true differential stress applied on the sample during the deformation can be easily calculated by dividing the applied force by the area on which it is applied [57]:

$$\sigma_d = \frac{IF}{A_{samplecorr}} \tag{S68}$$

To assess the reliability of the computed data, the error propagation analysis is performed following equation (S1) described by Taylor [59]:

$$\delta A_{samplecorr} = \sqrt{\left(\frac{\partial A_{samplecorr}}{\partial A_{sample}} \delta A_{sample}\right)^2 + \left(\frac{\partial A_{samplecorr}}{\partial \varepsilon_a} \delta \varepsilon_a\right)^2 + \left(\frac{\partial A_{samplecorr}}{\partial \varepsilon_v} \delta \varepsilon_v\right)^2} \tag{S69}$$

becoming:

$$\delta A_{samplecorr} = \sqrt{\left(\frac{(1-\varepsilon_v)}{(1-\varepsilon_a)} \delta A_{sample}\right)^2 + \left(A_{sample} \frac{(1-\varepsilon_v)}{(1-\varepsilon_a)^2} \delta \varepsilon_a\right)^2 + \left(\frac{A_{sample}}{(1-\varepsilon_a)} \delta \varepsilon_v\right)^2} \tag{S70}$$

which for the case $\varepsilon_v = 0$ is:

$$\delta A_{samplecorr} = \sqrt{\left(\frac{1}{(1-\varepsilon_a)} \delta A_{sample}\right)^2 + \left(A_{sample} \frac{1}{(1-\varepsilon_a)^2} \delta \varepsilon_a\right)^2} \tag{S71}$$

The error propagation analysis for the differential stress applied on the rock specimen is now straight forward. Similarly, to the error analysis for the corrected sample area, the error propagation for the applied differential stress results in:

$$\delta\sigma_d = \sqrt{\left(\frac{\partial\sigma_d}{\partial IF}\delta IF\right)^2 + \left(\frac{\partial\sigma_d}{\partial A_{samplecorr}}\delta A_{samplecorr}\right)^2} \tag{S72}$$

which becomes:

$$\delta\sigma_d = \sqrt{\left(\frac{1}{A_{samplecorr}}\delta IF\right)^2 + \left(-\frac{IF}{A_{samplecorr}{}^2}\delta A_{samplecorr}\right)^2} \tag{S73}$$

Permeability computation

To estimate the permeability of the sample under high temperature and confining pressure, the pore pressure oscillation method is used [22,26,56,65-68]. On the upstream reservoir a pressure oscillation with imposed frequency and amplitude is produced with a pump and the sample response is measured on a downstream pressure transducer (Supplementary Figure S3). The upstream oscillation amplitude may be chosen to be <10% of the equilibrium pore fluid pressure to avoid complex poroelastic effects [68].

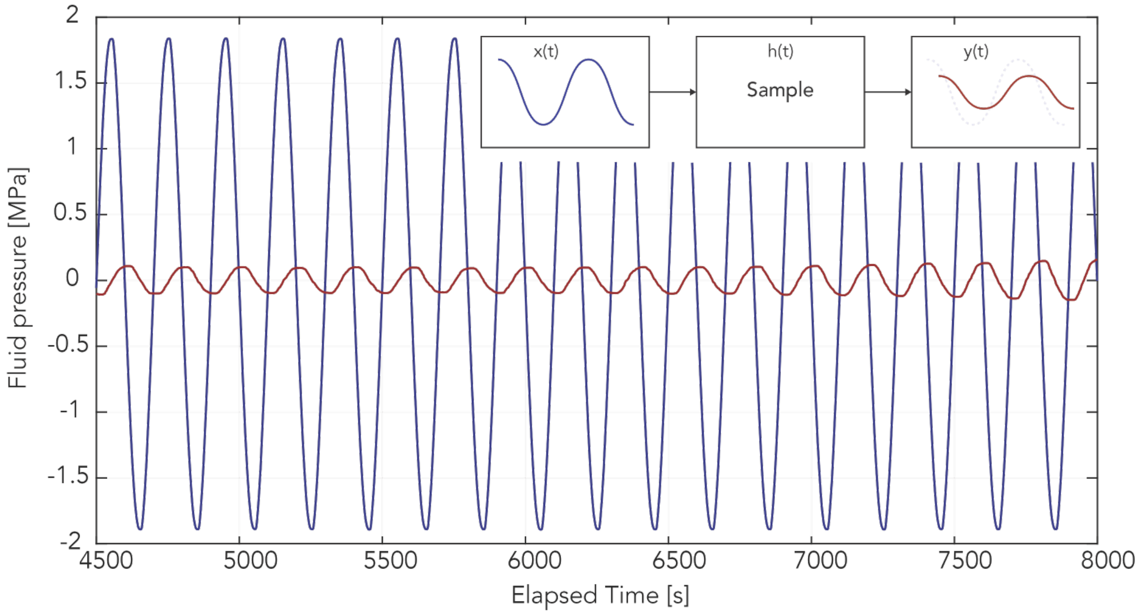

Supplementary Figure S3: Fluid pressure input and output signal during the pore pressure oscillation method. The blue signal is the upstream fluid pressure (input), whereas the red signal is the downstream fluid pressure (output). The pressure oscillations are represented as variation around the background fluid pressure value.

The downstream signal is characterised by a different amplitude (A_D) and a different phase (ϕ_D), with respect to the upstream pressure signal (A_U, ϕ_U). These differences in the output signal carry information on storativity and permeability of the sample [22,56,65,68], and are mainly controlled by the physical properties of the rock specimen, the fluid characteristics, and experimental setup [69]. The amplitude ratio $A = A_D/A_U < 1$ and the phase shift $\phi = \phi_D - \phi_U > 0$ are then used to estimate the permeability and storativity of the rock sample. Since in

our case the volume of the sample pore space is relatively small with respect to the volume of the downstream reservoir, storativity cannot be computed accurately and is therefore not considered in this work [65].

From the recorded upstream and downstream pressure data, only the oscillations are considered, therefore cutting and filtering the recorded data may be necessary to provide good input data to the processing routine. Early cycles may not be considered during the processing of the data, since early time transient effects may be present and affect the amplitude ratio and the respective phase shift [65]. For such reason, Bernabé et al. [65] suggest to record the pressure signals for several tens of cycle for static measurements. For measurements carried out during continuous deformation, however, frequency and amplitude of the input signal have to be chosen carefully in order to have enough oscillations to accurately capture permeability changes for small deformations. Since oscillations and deformation are continuous, to retrieve a continuous permeability, a moving window of several cycle is applied (in our case a widow of 10 cycle was used). The resulting permeability is an averaged value over an averaged deformation interval. As long as the changes in deformation, and therefore in permeability, are small enough compared to the time needed to retrieve enough data to estimate permeability, a continuous permeability measurement can be done under continuous deformation [19]. The oscillation frequency and amplitude have, therefore, to be chosen such that enough oscillation are present in a small deformation interval to gain an accurate estimate.

From the pressure data (upstream and downstream), the direct current signal (DC) is subtracted to avoid having high amplitude at 0 Hz in the frequency domain:

$$P_f^U = P_f^U - \overline{P}_f^U \tag{S74}$$

$$P_f^D = P_f^D - \overline{P}_f^D \tag{S75}$$

where subscript f stays for fluid, superscript U, D for upstream and downstream. The DC signal is represented by the mean of the pressure signal $\left(\overline{P}_f^{U,D}\right)$, which is constant and is the fluid pressure at which the experiment was performed.

Once the DC is removed, to obtain amplitude and phase from the recorded pressure signal, the discrete time series are transformed in the frequency domain by means of the Fast Fourier Transform (FFT). After having computed the single sided amplitude spectra for the upstream and downstream signal, the respective amplitude and phase can be retrieved from the transformed data. Afterwards the amplitude ratio A and phase shift ϕ can be computed. It is important to note that seen the periodicity of such functions, the resulting phase shift retrieved by the FFT could result to be a multiple of a cycle (2π), thus the necessity to reduce it eventually to a single cycle. Furthermore, phase shifts of more than one cycle should not be allowed during the experiment, since this can introduce errors in the analysis of the phases during its computation. Following Bernabé et al. [65] a non-dimensional permeability η and storage capacity ξ can be computed by solving the highly non-linear equation:

$$Ae^{(-i\phi)} = \frac{1}{\left(\frac{1+i}{\sqrt{\xi\eta}}\right)\sinh\left[(1+i)\sqrt{\frac{\xi}{\eta}}\right] + \cosh\left[(1+i)\sqrt{\frac{\xi}{\eta}}\right]} \tag{S76}$$

where

$$\eta = \frac{A_{sample} T k}{\pi l \mu \beta_D} \tag{S77}$$

$$\xi = \frac{A_{sample} l \beta}{\beta_D} \tag{S78}$$

with A_{sample} the sample cross-section area, l the sample length, T the oscillation period, k the sample permeability, μ the fluid viscosity, β_D the downstream reservoir storage capacity, and β the sample storativity.

To solve the above equation an iterative, gradient-based optimization method can be used. In a first stage, initial guesses (ξ_0, η_0) for the dimensionless permeability and storage capacity are made and values of A and ϕ are computed with help of equation (S76). Solving such non-linear equation is very sensitive to the choice of initial guesses. For such reason, a pre-search technique, which allows to obtain best estimates for the initial guesses, is applied [70]. In this method several initial guesses ξ_0 and η_0 are defined initially, amplitude ratio and phase shift are then computed with equation (S76) and the objective function or mismatch function (eq. S81) is subsequently evaluated. The initial guesses which have resulted in the objective function with the lower value are then used to solve equation (S76), ensuring accurate and fast convergence of the gradient-based optimization method.

The equation to be solved (eq. S76) is a complex valued equation, which can be split into two real equations, namely the real and imaginary part of it, thus forming two distinct equations that can easily be solved. The complex value to be solved for, $Ae^{(-i\phi)}$, is in polar coordinates, thus making it easy to split into two real equations. The amplitude ratio can also be expressed as the absolute value of the complex value:

$$A = abs\left(Ae^{(-i\phi)}\right) \tag{S79}$$

whereas the phase shift can be expressed as the angle in radians of the complex value that has to be found:

$$\phi = -angle\left(Ae^{(-i\phi)}\right) \tag{S80}$$

Once values for A and ϕ are found, they are compared with the amplitude ratio and phase shift retrieved during the experiment by evaluating the mismatch function [65]:

$$C(\xi_i, \eta_i) = w\left[\frac{\log_{10}(A_i)}{\log_{10}(A)} - 1\right]^2 + (1-w)\left[\phi_i - \phi\right]^2 \tag{S81}$$

where w is a weighting factor that can be chosen between 0 and 1 (usually, and in our case set to 0.5 [65]), ξ_i and η_i are the non-dimensional storage capacity and permeability at each iteration i. A_i and ϕ_i are the amplitude ratio and phase shift computed at each iteration with equation (S76). To minimise equation (S81) the gradient of the mismatch function is followed downward and ξ_i and η_i are update at each iteration:

$$x_{i+1} = x_i - \alpha_i \nabla C(\xi_i, \eta_i) \tag{S82}$$

with $\nabla C(\xi_i, \eta_i)$ being the gradient of the mismatch function evaluated at iteration i with A_i and ϕ_i, x_i the vector containing the searched variables ξ_i and η_i, and α_i the step size (<1) used to follow the gradient downward. While larger values of step size usually help to faster convergence but less accuracy, small step size results in more accuracy but slower convergence. The choice of the step size is crucial and can determine velocity and convergence of the gradient based descent method. For this reason, a small initial step size is chosen and then adapted at each iteration using the Barzilai-Borwein method [71,72] resulting in fast and accurate convergence of the iterative procedure:

$$\alpha_i = \frac{Y^T S}{Y^T Y} \tag{S83}$$

where $S = x_i - x_{i-1}$, $Y = \nabla C(\xi_i, \eta_i) - \nabla C(\xi_{i-1}, \eta_{i-1})$ and the superscript T indicates the transpose of the matrix.

Alternatively, it is also possible to retrieve values for non-dimensional permeability and storativity graphically from Supplementary Figure S4.

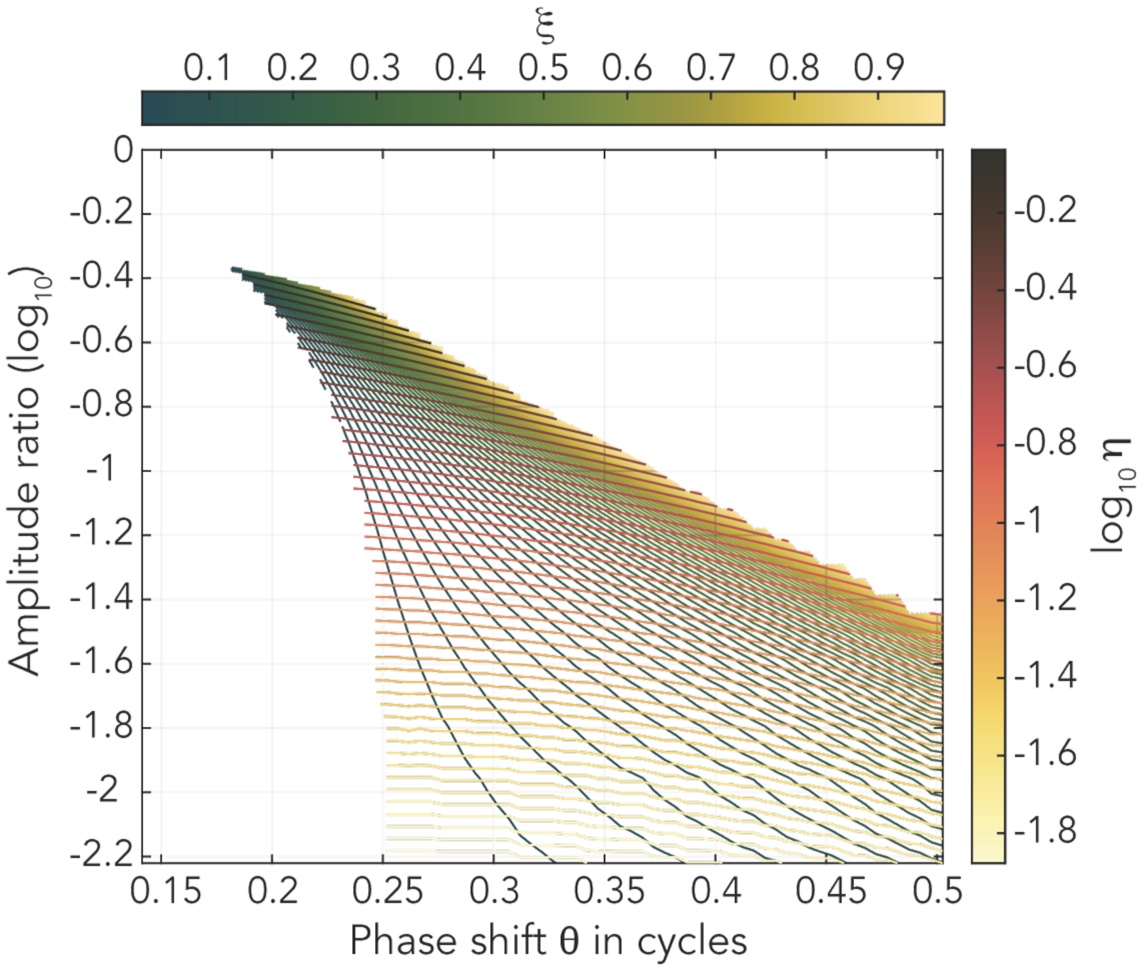

Supplementary Figure S4: Non-dimensional permeability and storativity for different phase shifts and amplitude ratios.

Once the non-dimensional permeability and storativity are retrieved, the actual specimen storativity and permeability can be determined:

$$k = \frac{\eta \pi l \mu \beta_D}{A_{sample} T} \quad \text{(S84)}$$

$$\beta = \frac{\xi \beta_D}{A_{sample} l} \quad \text{(S85)}$$

Bernabé et al. [65] express also how to compute the uncertainty in the measurements of amplitude ratio and phase shift. This method, however, is only easily applicable for static measurements, since it requires several oscillations at constant permeability, i.e. without deformation or changing conditions. For simplicity we assume that the computed phase shift and amplitude ratio have no uncertainty and so we do also assume no uncertainty for the computed non-dimensional permeability and storativity as well as for fluid viscosity, its bulk modulus and the oscillation period. Even with such assumptions an uncertainty can be computed following Taylor [59] for the permeability and storativity of the investigated sample, taking into account uncertainty in sample dimensions and downstream reservoir storage capacity:

$$\delta k=\sqrt{\left(\frac{\partial k}{\partial A_{sample}}\delta A_{sample}\right)^2+\left(\frac{\partial k}{\partial l}\delta l\right)^2+\left(\frac{\partial k}{\partial \beta_D}\delta \beta_D\right)^2} \quad \text{(S86)}$$

$$\delta \beta=\sqrt{\left(\frac{\partial \beta}{\partial A_{sample}}\delta A_{sample}\right)^2+\left(\frac{\partial \beta}{\partial l}\delta l\right)^2+\left(\frac{\partial \beta}{\partial \beta_D}\delta \beta_D\right)^2} \quad \text{(S87)}$$

turning into:

$$\delta k=\sqrt{\left(-\frac{\eta\pi\mu l\beta_D}{A_{sample}^2 T}\delta A_{sample}\right)^2+\left(\frac{\eta\pi\mu\beta_D}{A_{sample}T}\delta l\right)^2+\left(\frac{\eta\pi\mu l}{A_{sample}T}\delta \beta_D\right)^2} \quad \text{(S88)}$$

$$\delta \beta=\sqrt{\left(-\frac{\xi\beta_D}{A_{sample}^2 l}\delta A_{sample}\right)^2+\left(-\frac{\xi\beta_D}{A_{sample}l^2}\delta l\right)^2+\left(\frac{\xi}{A_{sample}l}\delta \beta_D\right)^2} \quad \text{(S89)}$$

References

59 Taylor, J. R. *An introduction to error analysis: the study of uncertainties in physical measurements*. 2nd edition edn, (Sausalito, California : University Science Books, 1997).

60 Paterson, M. *HPT Machine Records*, <https://openresearch-repository.anu.edu.au/handle/1885/117174> (1994-2010).

61 Covey-Crump, S. J., Xiao, W. F., Mecklenburgh, J., Rutter, E. H. & May, S. E. Exploring the influence of loading geometry on the plastic flow properties of geological materials: Results from combined torsion + axial compression tests on calcite rocks. *Journal of Structural Geology* **88**, 20-31, doi:10.1016/j.jsg.2016.04.007 (2016).

62 Buffington, F. S., Hirano, K. & Cohen, M. Self diffusion in iron. *Acta Metallurgica* **9**, 434-439, doi:10.1016/0001-6160(61)90137-7 (1961).

63 La Rochelle, P., Leroueil, S., Trak, B., Blais-Leroux, L. & Tavenas, F. in *Advanced Triaxial Testing of Soil and Rock* Vol. 28 (eds Robert T. Donaghe, Ronald C. Chaney, & Marshall L. Silver) 715-731 (American Society for Testing and Materials, ASTM STP 977, 1988).

64 Omar, T. & Sadrekarimi, A. Effects of Multiple Corrections on Triaxial Compression Testing of Sands. *Journal Of Geoengineering* **9**, 75-83, doi:10.6310/jog.2014.9(2).3 (2014).

65 Bernabé, Y., Mok, U. & Evans, B. A note on the oscillating flow method for measuring rock permeability. *International Journal of Rock Mechanics and Mining Sciences* **43**, 311-316, doi:https://doi.org/10.1016/j.ijrmms.2005.04.013 (2006).

66 Song, I. & Renner, J. Analysis of oscillatory fluid flow through rock samples. *Geophysical Journal International* **170**, 195-204, doi:10.1111/j.1365-246X.2007.03339.x (2007).

67 Yang, D., Wang, W., Chen, W., Tan, X. & Wang, L. Revisiting the methods for gas permeability measurement in tight porous medium. *Journal of Rock Mechanics and Geotechnical Engineering* **11**, 263-276, doi:https://doi.org/10.1016/j.jrmge.2018.08.012 (2019).

68 Kranz, R. L., Saltzman, J. S. & Blacic, J. D. Hydraulic diffusivity measurements on laboratory rock samples using an oscillating pore pressure method. *International Journal of Rock Mechanics and Mining Sciences & Geomechanics Abstracts* **27**, 345-352, doi:https://doi.org/10.1016/0148-9062(90)92709-N (1990).

69 Falcon-Suarez, I. & Canal-Vila, J. *Techniques for rock permeability determination II. The pore pressure oscillation method.* (2013).

70 Wicklin, R. *How to find an initial guess for an optimization (access date 2019.06.23)*, <https://blogs.sas.com/content/iml/2014/06/11/initial-guess-for-optimization.html> (2014).

71 Barzilai, J. & Borwein, J. M. Two-Point Step Size Gradient Methods. *IMA Journal of Numerical Analysis* **8**, 141-148, doi:10.1093/imanum/8.1.141 (1988).

72 Zhang, H. & Mandic, D. P. Is a Complex-Valued Stepsize Advantageous in Complex-Valued Gradient Learning Algorithms? *IEEE Transactions on Neural Networks and Learning Systems* **27**, 2730-2735, doi:10.1109/TNNLS.2015.2494361 (2016).
